# Refuting the hypothesis that the acquisition of germ plasm accelerates animal evolution

**DOI:** 10.1038/ncomms12637

**Published:** 2016-08-31

**Authors:** Carrie A. Whittle, Cassandra G. Extavour

**Affiliations:** 1 Department of Organismic and Evolutionary Biology, Harvard University, 16 Divinity Avenue, Cambridge, Massachusetts 02138, USA; 2 Department of Molecular and Cellular Biology, Harvard University, 16 Divinity Avenue, Cambridge, Massachusetts 02138, USA

## Abstract

Primordial germ cells (PGCs) give rise to the germ line in animals. PGCs are specified during embryogenesis either by an ancestral mechanism of cell–cell signalling (induction) or by a derived mechanism of maternally provided germ plasm (preformation). Recently, a hypothesis was set forth purporting that germ plasm liberates selective constraint and accelerates an organism's protein sequence evolution, especially for genes from early developmental stages, thereby leading to animal species radiations; empirical validation has been claimed in vertebrates. Here we present findings from global rates of protein evolution in vertebrates and invertebrates refuting this hypothesis. Contrary to assertions of the hypothesis, we find no effect of preformation on protein sequence evolution, the evolutionary rates of early-stage developmental genes, or on species diversification. We conclude that the hypothesis is mechanistically implausible, and our multi-faceted analysis shows no empirical support for any of its predictions.

## Abstract (web summary)

The evolution of germ cell specification by maternal germ plasm has been proposed to accelerate vertebrate protein evolution by liberating selective constraints. Whittle and Extavour analyse global rates of protein evolution and find no support for this hypothesis in vertebrates or invertebrates.

## Graphical abstract

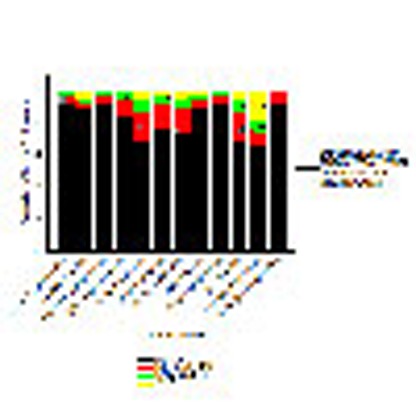

PGCs in animals typically form by one of two modes: first, the evolutionarily conserved mode known as induction (sometimes called epigenesis^1^), wherein PGCs are induced from presumptive mesoderm in the embryo; or second, the derived mode known as preformation (sometimes called inheritance), wherein PGCs are determined by preformed germ plasm inherited by maternal or early embryonic tissues^1^,^2^. On the basis of the phylogenetic distribution of these mechanisms across metazoans, induction is thought to be the ancestral animal mode of PGC specification, with preformation having arisen convergently multiple times in various animal phyla^1^,^3^. However, the selective pressures that could favour repeated evolution of the preformation mode are a matter of current debate. A recent hypothesis (referred to hereafter as the PGC-specification hypothesis) claims that preformation accelerates evolution as compared to induction^2^,^4^,^5^,^6^. This hypothesis posits that in organisms with induction, the requirement for induction of PGCs by neighbouring somatic cells, would act as a constraint on the early embryonic somatic tissues, and ultimately the fates and morphogenesis of an organism's somatic gene networks including those involved in late embryos and postembryonic stages^4^,^5^. In turn, under preformation, distinguishing somatic from germ line fates at the onset of development or even before fertilization would liberate constraint on genes and cellular behaviours involved in somatic tissue specification, patterning and morphogenesis^6^. This hypothesis thus predicts that organisms with preformation should exhibit enhanced ‘evolvability' of proteins and morphology, as compared with animals ‘constrained' under the induction mode^2^,^6^.

The PGC-specification hypothesis has several predictions, each of which has profound consequences for animal evolutionary biology. First, a central prediction of the hypothesis is that preformation leads to elevated rates of changes in proteins, at a level that is observable at a genome-wide level (suggested to be up to 32% of the protein-coding sequences in a taxon^6^). Accordingly, this would mean that PGC-specification mode is a major factor shaping the evolution of coding-DNA, and thus crucial to our understanding of how animal genomes evolve. A secondary facet of this hypothesis is that the rapid evolution of proteins under preformation is most pronounced for genes expressed in early embryogenesis as compared with later developmental stages^6^, since major tissue types are specified, patterned and shaped largedly at early stages of development. This would mean that PGC-specification mode is also an essential contributor to the evolution of early developmental genes. Finally, the hypothesis predicts that the proposed liberation of selective constraint under preformation leads to freedom to evolve diverse morphologies (evolvability), and thus markedly enhances species radiations^6^, a concept suggested to be supported by observations of elevated species richness in some vertebrate clades with preformation as compared with clades with induction^2^,^5^. Under this scenario, PGC-specification mode would be a predominant factor contributing to the evolution of new species throughout animal evolutionary history. Taken together, the PGC-specification hypothesis, if well supported, could have widespread implications in genome biology and evolution.

The only empirical study to date testing this hypothesis was recently conducted among four pairs of divergent vertebrates, with one member of each pair displaying preformation, and the other displaying induction (anurans versus urodeles, birds versus crocodiles/turtles, snakes versus lizards and one clade of ray-finned fishes (Teleostei) versus another (Acipenseriformes))^6^. However, that study had notable limitations. First, rapid protein evolution, as inferred from incongruent gene trees, was observed for the preformation lineage (as compared with induction) for only two of the four main taxon contrasts. Second, protein evolution was studied using only first and second codon positions and third nucleotide positions of codons were excluded from the analysis due to saturation, since the clades being compared were too divergent in genome sequence to allow inclusion of the third codon position. As a change at the first and second positions of codons nearly always results in an amino-acid change (based on the genetic code, a change at the second position always, and at the first position usually (96%), causes an amino-acid substitution^7^), analysing only these two codon positions yields a statistic that loosely reflects the nonsynonymous substitution rate (dN). However, this approach cannot provide information on the synonymous substitution rate (dS; silent changes), nor most importantly about selection, which requires the ratio dN/dS^8^,^9^. By excluding dS (and thus dN/dS), one cannot ascertain whether observations of high dN result from an elevated mutation rate, and thus neutral evolution in a lineage, or from the liberation of selective pressures^8^,^9^,^10^. Third, the taxa used for each sequence analyses (for example, birds, crocodiles, mammals and an outgroup), were massively divergent, causing saturation, potentially making sequence alignments and substitution rate estimates unreliable^11^. Fourth, the assessment included many paired contrasts of preformation and induction species that were not phylogenetically independent. As an example, a large number of overlapping contrasts of anuran species versus urodele species were treated as independent data points, an approach known to cause tenuous correlations due to pseudoreplication^12^. Moreover, some of the species chosen only had substantially fewer than 500 partial-coding regions available for study, which does not represent a substantial part of the genome, and were derived from expression data sets from particular tissues, likely causing biases towards certain types of genes or functions (for example, brain, gonads and venom). Finally, invertebrates, which comprise over 97% of animals on earth^13^, were excluded from analysis. Thus, it remains unknown whether the hypothesis of rapid protein evolution across a major portion of the genome under preformation holds for a broad range of animals, under analyses not limited by these methodological caveats. Moreover, the secondary facets of this hypothesis, namely the notion that preformation accelerates evolution of early-expressed developmental proteins as compared with those expressed at later stages, and that preformation promotes animal speciation, each warrant further evaluation.

Here based on comparative molecular evolutionary analysis in a wide range of animals, we show that the PGC-specification hypothesis is evolutionarily improbable, and that our empirical analysis provides no evidence in favour of any of its predictions.

## Results

### Preformation does not affect protein sequence evolution

**Figure 1:**
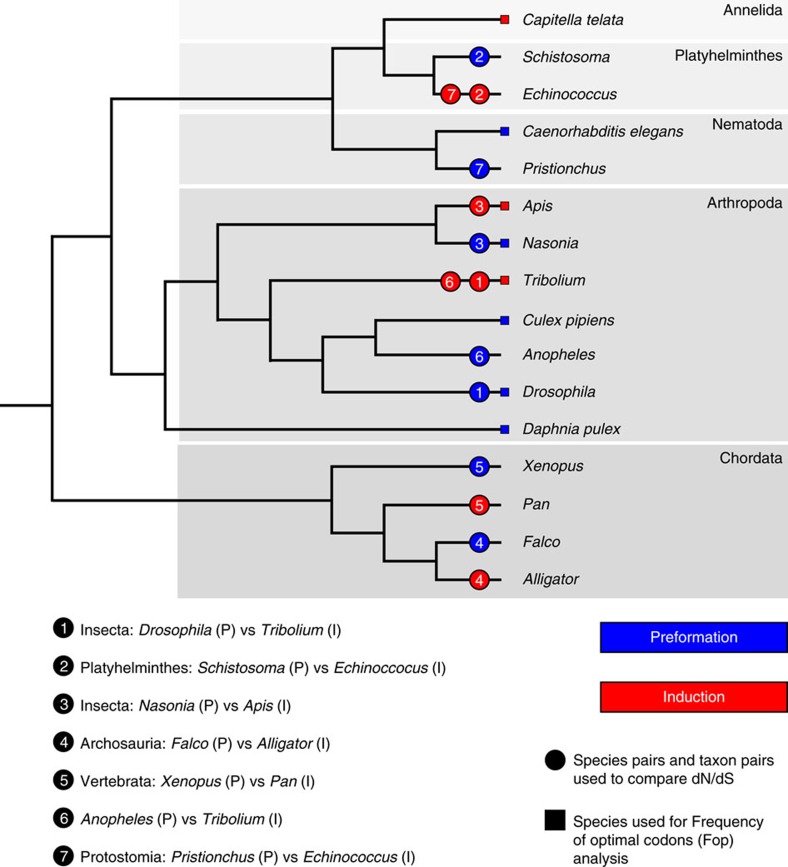
The phylogenetic relationships among vertebrate and invertebrate taxa analysed. The mode of PGC formation (preformation (P): blue, induction (I): red) is shown on the branches. With respect to dN/dS, 12 genera were studied (two species per genus), and thus all comprise independent data points. In addition, the 12 genera were grouped into paired comparisons: pairs 1–5 represent phylogenetically independent contrasts (no overlap in the phylogeny between contrasts) and 6–7 are supplementary (non-independent) contrasts (Table 1).

For our analyses, we assessed whether preformation, but not induction, correlated with accelerated protein sequence evolution in a manner detectable across the genome in animals, including vertebrates and invertebrates. The PGC-specification mode does not typically vary within a single genus/family in animals (Fig. 1); this impedes common methods such as contrasts of dN/dS among species with preformation and induction mode across a single phylogeny due to saturation^9^, but does differ between genera within a phylum (Fig. 1). Here we measured dN/dS between orthologues for pairs of species within the same genus for taxa with preformation and induction modes^14^ as shown in Fig. 1 and Table 1. Genera were chosen based on strong cytological or experimental support for the mode of PGC-specification mode (Supplementary Table 1), availability of whole-genome sequence data for two species within the same genus (Supplementary Tables 2 and 3), and whenever possible, a second genus from the same phylum matching these criteria with an opposite PGC-specification mode. Using these criteria, we identified 12 animal genera for study: the invertebrate genera *Drosophila*; *Tribolium*; *Schistosoma*; *Echinococcus*; *Nasonia*, *Apis*; *Anopheles*; and *Pristionchus*, and the vertebrate genera *Falco*, *Alligator*, *Xenopus* and *Pan*. As dN/dS was determined between pairs of species within a genus, each of these 12 genera comprises an independent data point that is comparable to all other genera^14^. As a secondary assessment, we grouped the genera into five non-overlapping phylogenetically independent intergeneric contrasts of closely related pairs with opposite PGC modes (preformation versus induction) from the same phylum (see ‘Primary dN/dS contrasts' Table 1; Fig. 1). We also included two supplemental contrasts (*Pristionchus* versus *Echinococcus*, and *Anopheles* versus *Tribolium*) with the important recognition that these were complementary tests (and not phylogenetically independent, and the former case spanned phyla) to our primary analysis. Given that all 12 within-genus species-pairs under study are closely related and independent, this approach avoids limitations of saturation, alignments across highly divergent taxa, and non-independence of contrasts^12^,^15^,^16^,^17^, while providing a signal of rates of protein evolution across the genome^14^. In additon, this approach measures the current/ongoing rates of divergence (between two species in a genus), and avoids the potential misleading influence of bursts of rapid evoution that could occur anywhere on the branch from the last ancestor, which could afflict studies performed with highly divergent organisms^6^.

**Figure 2:**
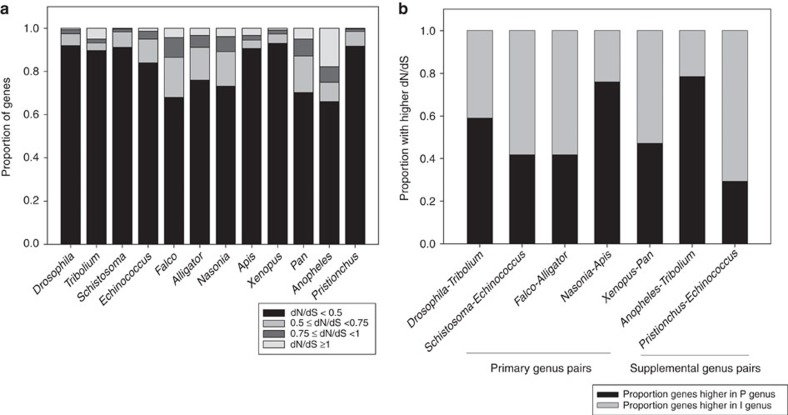
The relationship between dN/dS and PGC-specification mode. (**a**) The profile of dN/dS for each vertebrate and invertebrate species pair per genus under study. Genes have been divided into four distinct dN/dS categories based on magnitude. (**b**) The proportion of orthologous genes with ⩾1.5-fold higher dN/dS in preformation versus induction genera for intergeneric contrasts. P, preformation; I, induction.

Analysis of dN/dS in the 12 genera provides no evidence that the preformation specification mode accelerates molecular evolution in these animals. Typically dN/dS<1, dN/dS=1 and dN/dS>1 indicate purifying selection, neutral evolution and positive selection, respectively^9^. Because whole-gene dN/dS ratios are conservative measures of selection, even when dN/dS<1, genes with elevated values suggest events of relaxed selection or adaptive evolution. CDS were placed into one of four bins based on magnitude of dN/dS (dN/dS<0.5, 0.5≤dN/dS<0.75, 0.75≤dN/dS<1 and dN/dS⩾1) as shown in Fig. 2a. As each of the 12 genera in Fig. 2a (within-genus species pairs) are independent data points, we compared the dN/dS profiles across all taxa. For all genera, including preformation and induction organisms (Fig. 1, Table 1), the vast majority of CDS had dN/dS values <0.5, consistent with strong purifying selection (Fig. 2a). Further, there was no tendency for more genes to evolve rapidly under preformation. For example, the six genera with the fastest evolving genomes (highest proportion of genes per genome with dN/dS>0.5) were *Echinococcus* (induction), *Falco* (preformation), *Alligator* (induction), *Nasonia* (preformation), *Pan* (induction) and *Anopheles* (preformation). As this sample contains three preformation and three induction taxa, it demonstrates that among those organisms with the highest proportion of genes with enhanced ‘evolvability', or dN/dS>0.5, there is not even a slight tendency (>50%) for the taxa to use the preformation mode, rather than induction, in these animals.

Marginal differences were observed in genome-wide dN/dS profiles between the genera in Fig. 2a; however, these were unrelated to preformation or induction modes in a consistent way. For example, in the invertebrate *Drosophila* (preformation) >90% of CDS had values <0.5 (also see ref. 18), nearly identical to its sister taxon *Tribolium* (induction). Further, a lower fraction of genes had dN/dS>1 in *Drosophila* (0.68%) than in *Tribolium* (5.0%), suggesting positive selection is more common under induction (Fig. 2a). Strikingly similar dN/dS profiles were observed between *Schistosoma* (preformation) and *Echinococcus* (induction), with a marginally higher level (7%) of genes with dN/dS>0.5 for the induction taxon, rather than the preformation taxon. *Nasonia* (preformation) exhibited an elevated level of CDS with relatively high dN/dS compared with its sister taxon *Apis* (induction), with 26.8% and 9.5% having dN/dS>0.5, respectively, but had a similar proportion of CDS with dN/dS>1. Collectively, dN/dS does not show any consistent relationship to PGC mode in these invertebrates.

Within vertebrates, a *Xenopus* (frog) versus *Ambystoma* (salamander) comparison is often invoked in discussion of PGC-specification mode due to strong evidence of preformation and induction modes, respectively^1^,^6^,^19^. However, the small data sets for the latter taxon used in Evans *et al*.^6^ were deemed unsuitable for study here (Methods). We therefore compared *Xenopus* (preformation) versus *Pan* (induction); although divergent chordates, a strong effect of preformation on protein evolution in *Xenopus*, as reported by Evans *et al*.^6^ should still be evident. We found four times as many genes in the induction genus had dN/dS>0.5 compared with the preformation genus, implying that if anything, induction is associated with accelerated protein sequence evolution. In fact, *Xenopus* had the highest percentage of dN/dS<0.5 (92.9%) among all 12 genera under study, consistent with the lowest level of evolutionary change (fewest fast-evolving proteins). The vertebrates *Falco* (preformation) and *Alligator* (induction) exhibited among the highest percentage of CDS (>24%) with dN/dS>0.5, suggesting both genera exhibit greater propensity for relaxed or positive selection than the other remaining genera (Fig. 2a). However, only a marginal difference (<8%) was observed in the fraction of CDS per genome with high dN/dS (>0.5).

Mann–Whitney *U* (MWU)-tests of genome-wide dN/dS per genus were statistically significantly different for four of the five between-genera pairs outlined in Table 1. The differences were as follows: *Drosophila*>*Tribolium*, *Schistosoma*<*Echinococcus*, *Nasonia*>*Apis*, *Xenopus*<*Pan* (*P*<10^−15^ for each contrast), with no difference for *Falco* and *Alligator* (*P*=0.13; Supplementary Note 1), thus showing no consistent effect of PGC-specification mode. Supplementary contrasts of *Anopheles* (preformation) versus *Tribolium* (induction) and *Pristionchus* (preformation) versus *Echinococcus* (induction) revealed the preformation and induction taxa respectively, evolved more rapidly (MWU-tests *P*<10^−15^; Fig. 2a; Supplementary Note 1), again showing no relevant effect of PGC-specification mode.

### Between-genus orthologues show no effect of preformation

Next we studied dN/dS among specific orthologues matched across five pairs of genera (Table 1, Fig. 1); we identified those orthologues with at least a 1.5-fold difference in dN/dS between the genus with induction and preformation (per between-genus pair). We found that dN/dS in the orthologous CDS sets was unrelated to PGC-specification mode. For instance, for *Nasonia* (preformation) and its sister taxon *Apis* (induction), 76.0% of the 2,161 orthologues exhibiting a ⩾1.5-fold difference in dN/dS between taxa had a higher value in the preformation taxon, which may appear consistent with more genes in this CDS subset evolving rapidly under preformation. However, for *Drosophila* (preformation) and *Tribolium* (induction), 58.9% of the 2,921orthologues with a ⩾1.5-fold difference had higher dN/dS under preformation), a difference level inconsistent with globally rapid CDS under preformation. Further, for *Schistosoma* (preformation) versus *Echinococcus* (induction), 58.2% of the orthologues with at least a 1.5-fold difference in dN/dS (*N*=1,321) had higher values in the induction taxon (Fig. 2b), not the preformation taxon. Altogether, these results in invertebrates, consistent with the findings across all genes (Fig. 2a), show no pattern with respect to PGC-specification mode and fail to support the prediction that germ plasm accelerates protein sequence divergence.

For vertebrates, the *Falco* (preformation) and *Alligator* (induction) contrast showed rapid evolution was more commonly observed under induction than preformation: 58.1% of the 2,537 CDS exhibiting >1.5-fold difference had elevated dN/dS for the induction taxon) The dN/dS values for the two *Falco* species (*F. cherrug* and *F. peregrinus*) correspond with prior findings for these taxa (mean dN/dS herein=0.36±6.2 × 10^−5^, mean therein 0.39), where it was shown they exhibit high dN/dS within the bird clade^20^. Despite having high dN/dS within birds, they still exhibit no notable elevation with respect to alligators (Fig. 2b). Bird genes have previously been found to exhibit lower (as well as higher), dN/dS than their orthologues in other induction taxa such as mammals, which largely depends on the ontology class^21^ and thus not PGC mode; further confirming no major role of PGC-specification mode in birds (Supplementary Note 2). In *Xenopus* (preformation) versus *Pan* (induction), 47.3% of orthologues with 1.5-fold difference (*N*=2,471) had elevated dN/dS under preformation, and 52.7% had higher values under induction (Fig. 2b), inferring marginally higher rates when genes evolve under induction. Altogether, the two vertebrate contrasts show no signal of rapid sequence divergence under preformation. The supplemental contrasts of *Anopheles* (preformation) versus *Tribolium* (induction) and *Pristionchus* (preformation) versus *Echinococcus* (induction) revealed that more genes evolved rapidly for the preformation and the induction taxon, respectively (Fig. 2b), and thus no effect of PGC-specification mode.

While we cannot exclude that species-specific factors obscure a mild PGC mode effect, it is evident that if preformation liberates selective constraint and broadly enhances protein sequence evolution in animals, we would expect a detectable signal from the 12 independent genera data points (Fig. 2a) and from the five paired between-genera contrasts (Fig. 2b). As discussed in Supplementary Note 3, we exclude an effect of divergence times, and population size on our results. In addition, it is important to note that since dN/dS was determined within genera, dS was well below saturation levels (<1) for all taxa under study herein, as shown in the bar and whisker plots provided in Supplementary Fig. 1. Thus, our collective results of dN/dS across genera in Fig. 2a,b show no pattern with respect to PGC-specification mode and fail to support the prediction that germ plasm accelerates protein sequence divergence.

As a complementary test to dN/dS, we assessed the frequency of optimal codons (Fop) relative to PGC-specification mode for various animals. Optimal codon usage has been employed in *Drosophila* and other eukaryotes to detect rapidly evolving proteins^22^,^23^,^24^, as proteins that evolve rapidly tend to have low Fop^22^,^23^,^24^,^25^,^26^. We identified or verified the optimal codon lists for the taxa in Supplementary Table 4, and subsequently examined Fop for the preformation taxa *Caenorhabditis elegans*, *Culex pipiens* and *Daphnia pulex* and the induction species *Capitella teleta* (Supplementary Note 4; Supplementary Tables 5 and 6; and Supplementary Figs 2 and 3). No notable trends departing from normality were observed in the distributions of Fop for all three preformation species (Supplementary Fig. 2), indicating no tendency for rapid protein evolution under preformation. Similarly, for *C. teleta* (induction), there were no notable trends toward high Fop in the distribution that would suggest a broad tendency for slowed protein evolution under induction (Supplementary Fig. 2).

### Preformation is unlinked to divergence of early-stage genes

**Figure 3:**
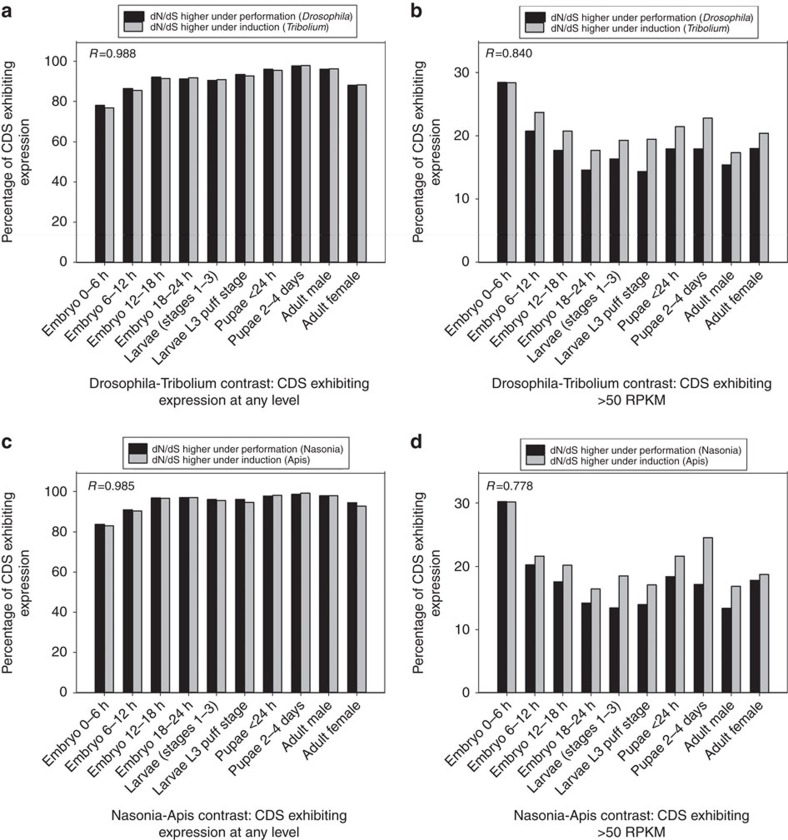
The developmental expression profiles of CDS with high dN/dS within the *Drosophila*–*Tribolium* and the *Nasonia*–*Apis* contrasts. (**a**) The percentage of the high dN/dS CDS set (⩾1.5-fold difference in dN/dS) for genera from *Drosophila* (preformation: black bars) and from *Tribolium* (induction: grey bars) expressed at each developmental stage, and (**b**) the per cent expressed at >50 RPKM at each developmental stage. (**c**) The percentage of the high dN/dS CDS set from *Nasonia* (preformation: black bars) and from *Apis* (induction: grey bars) expressed at each developmental stage, and (**d**) the per cent expressed at >50 RPKM at each developmental stage. Spearman correlations (*R*) among the preformation and induction taxa across developmental stages are shown (*P*<2.0 × 10^−7^ for all *R* values). Note 2 of the 10 stages/data points were adult males and females.

A second facet of the PGC-specification hypothesis is that preformation releases selective constraint more frequently in genes expressed at early embryogenesis, as compared with later developmental stages, a phenomenon not inherent to induction; this has been purported to be empirically supported in vertebrates^6^. In that assessment, the authors identified CDS with high dN in any of the preformation taxa studied, asked when the mouse or zebrafish orthologues of these genes were expressed during embryogenesis, and asserted that the orthologues were mainly expressed in early stages of development. However, no comparable assessment was conducted for genes that appeared to evolve rapidly in induction taxa. Here we investigated expression of all identifiable orthologues in the *Drosophila*–*Tribolium* and *Nasonia*–*Apis* contrasts (preformation-induction, respectively), which were the two (out of five) between-genus pairs with some sign of elevated dN/dS under preformation (Fig. 2b). There were two CDS sets per contrast: the set with 1.5-fold higher dN/dS in the preformation taxon and the non-overlapping set with 1.5-fold higher dN/dS (referred to hereafter as high dN/dS CDS sets) in the induction taxon. Using the comprehensive developmental expression database in *Drosophila* (Methods; http://www.flybase.org (ref. 27), the expression profile of the high dN/dS CDS were examined across 10 developmental stages/phases from 0 to 6 h embryos up until adulthood (Fig. 3).

In *Drosophila*, we found a lower percentage (78.0%) of the high dN/dS CDS set was expressed in 0–6 h embryos than in all nine later developmental stages (between 86.4 and 97.7%; *χ*^2^ *P*<0.001 for all paired contrasts), inconsistent with preferential expression of fast-evolving CDS in early developmental stages under preformation. Further, the high dN/dS set from *Drosophila* (preformation) and its counterpart in *Tribolium* (induction) had nearly identical profiles with respect to development (Fig. 3a; the difference was <1.2% for each of 10 developmental stages (*χ*^2^ *P*>0.63) and the percentages across stages were highly correlated between genera (Spearman's *R*=0.985, *P*<2 × 10^−7^; Supplementary Note 5).

For further stringency, we asked whether the high dN/dS CDS in *Drosophila* (preformation) were more commonly expressed at elevated levels (>50 reads per kilobase million (RPKM); defined as ‘high' expression based on the whole transcriptome in Flybase, http://www.flybase.org) in early embryos as compared to the set from *Tribolium* (induction), as these genes may be most apt to be linked to crucial functions. Within the high dN/dS CDS from *Drosophila* and from *Tribolium*, the 0–6 embryos each exhibited a mildly (maximum of 14.0% difference) greater percentage of CDS with >50 RPKM (28.4%, in the 0–6 h embryos in both *Drosophila* and *Tribolium*), than the nine later developmental stages, with values between 14.4 and 23.7% (Fig. 3b; *χ*^2^ *P*<0.001 for each contrast per taxon). The proportions, however, were in effect identical for the preformation and the induction taxa for 0–6 h embryos (*χ*^2^ *P*=1, Fig. 3b), and were highly correlated between taxa across all developmental stages (Spearman's *R*=0.840, *P*<2.0 × 10^−7^), and thus disagree with the PGC-specification hypothesis.

The second independent assessment on the *Nasonia* (preformation) and *Apis* (induction) contrast yielded virtually identical results. For *Nasonia*, a lower percentage of high dN/dS CDS (>1.5-fold higher dN/dS in *Nasonia*) were expressed in 0–6 h embryos (83.7%) than all later stages (90.9 to 98.6%, *χ*^2^ *P*<0.001 for all contrasts, Fig. 3c). The proportions of the *Nasonia* and *Apis* high dN/dS CDS sets expressed at each stage were nearly identical (<1.7% difference across all stages, *χ*^2^ *P*>0.19 for all contrasts, Fig. 3c) and highly correlated (Spearman's *R*=0.985, *P*<2.0 × 10^−7^). In turn, the proportion of high dN/dS CDS with >50 RPKM in 0–6 h embryos was nearly identical between *Nasonia* and *Apis*, (30.2% and 30.1%, respectively, *χ*^2^ *P*=0.98), and values highly correlated across development between genera (*R*=0.778, *P*<2.0 × 10^−7^, Fig. 3d).

Collectively, neither of the *Drosophila*–*Tribolium* or *Nasonia*–*Apis* contrasts, the only two contrasts (of five main between-genus contrasts, Table 1) that showed some tendency for more genes to evolve rapidly under preformation (Fig. 2b), support the notion that fast-evolving genes under preformation are preferentially linked to early development. We therefore conclude that at least for these two pairwise comparisons of induction versus preformation taxa: (1) fast-evolving genes under preformation are not linked to early development; and (2) developmental expression profiles of fast-evolving genes are nearly identical under preformation versus induction.

### Developmental genes

**Figure 4:**
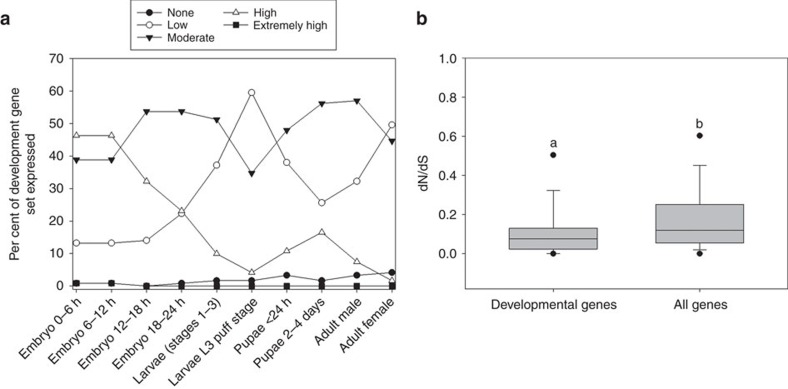
The dN/dS of developmental genes in *Drosophila*. (**a**) The percentage of the 121 developmental genes with no, low, moderate, high or extremely high expression at each of ten stages of development in *Drosophila*. (**b**) Box and whisker plots (showing the 25th percentile/median/75th percentile and whiskers representing maximum and minimum excluding outliers) of dN/dS for the developmental genes and all remaining genes in *Drosophila* (*D. melanogaster* and *D. simulans*) genome. Different letters indicate a statistically significant difference using the MWU-test (*P*<0.001). Expression levels for (**a**) are as follows: none (0 RPKM), low (>0 RPKM ≤10), moderate (>10 RPKM ≤50), high (>50 RPKM ≤1,000) or extremely high (>1,000).

As preformation has been proposed to release constraint on development and allow greater morphological variation that could contribute to speciation^2^, we assessed evolutionary rates of developmental genes. We chose genes that are known to play important roles in the development in animals, have well-supported annotations, known functions, expression profiles and complete CDS (without unknown sites) in the model *D. melanogaster* (flybase.org), and with well-defined orthologues in *D. simulans* to allow assessment of dN/dS. Using these criteria, we identified 121 developmental genes for analysis (Supplementary Table 7). As shown in Fig. 4a, all 121 studied genes were expressed in at least one developmental stage, and >95% of this gene set was expressed all developmental stages. The average dN/dS for this developmental gene set was 0.118±0.014 (median of 0.076), which was statistically significantly lower than for the remainder of CDS in the genome (Average=0.189±0.002, MWU-test *P*<0.001, Fig. 4b), indicating strong purifying selection, as may be expected for genes involved in crucial and multi-stage functions^28^,^29^,^30^. Further, no differences were detected in dN/dS of the matching putative orthologues between *Drosophila* (preformation) and the *Tribolium* (induction) genus (MWU-test *P*>0.55). In summary, we extend our conclusions that preformation does not enhance dN/dS of CDS at levels detectable across the genome (Fig. 2a,b), including CDS expressed at early stages (Fig. 3a–d), to also include genes specifically involved in development (Fig. 4a,b).

## Discussion

The collective results herein do not support the hypothesis that the acquisition of germ plasm accelerates animal evolution. First, the 12 independent within-genus estimates of genome-wide dN/dS (Fig. 2a), as well as five paired intergeneric contrasts of matched orthologues in taxa with distinct PGC-specification modes (Fig. 2b), failed to support the assertion that germ plasm causes accelerated protein divergence (high dN/dS). If germ plasm broadly released morphological and sequence constraint^2^,^6^, all preformation taxa studied herein, including the vertebrate taxa suggested by Evans *et al*.^6^, should have exhibited fast rates of evolution in protein-coding genes across the genome (Fig. 2a,b). Instead, we observed not even a slight tendency in favour of this hypothesis: preformation and induction taxa were equally represented among the six genera with the fastest evolving genomes (Fig. 2a), and in the paired between-genus contrasts, fewer than half of the preformation genera showed any inclination for genes to evolve more rapidly than in induction genera (Fig. 2b). Second, our findings that early developmental genes were not evolving rapidly under preformation, and that the more rapidly evolving genes had nearly identical developmental expression profiles in both preformation and induction taxa (Fig. 3a–d), also counter the PGC-specification hypothesis of Evans *et al*.^6^. Importantly, although the Evans *et al*. hypothesis addresses only vertebrate evolution, our analyses provide no support for this hypothesis in either vertebrates or invertebrates.

A third facet of the PGC-specification hypothesis is that the acquisition of germ plasm, and fast evolution of protein-sequences, leads to enhanced speciation^2^,^6^. Anecdotal data based on species richness in vertebrate clades has been taken as support for this proposal^2^,^6^. For instance, it has been contended that the much higher number of species in some vertebrate clades with preformation, such as frogs (number of species estimated as 4,800), ascidians (3,000), teleosts (25,000) and birds (10,000), than in other groups with induction, including turtles (300), lancelets (23), non-teleost actinopterygians (44), salamanders (515) and hemichordates (100) provides evidence of higher speciation rates^2^. However, a rigorous assessment of species diversification rates would require large-scale phylogenetic data sets and multi-faceted intensive techniques, including assessments of clade-age and birth-deaths, approaches which are still largely under development, testing and refinement^31^,^32^,^33^. Methodological or data set challenges notwithstanding, anecdotal examples of species richness alone cannot be used to make strong conclusions about speciation rates.

Acknowledging that species-richness alone^2^,^6^ comprises a relatively weak non-analytical approach to assessing diversification rates with respect to PGC-specification mode^34^, even if one uses that approach, there are many anecdotal examples in the literature that support the opposite trend, of large radiations under induction. For instance, mammals (induction, 5,400 species^2^) and lizards (induction, >6,100 species; www.reptile-database.org) also exhibit high levels of diversification. Further, an available diversification-rate assessment based on clade-age and birth-death analysis from 44 clades of jawed-vertebrates suggests despite their high species richness, frogs (preformation) do not exhibit an elevated (non-typical) diversification rate in this taxonomic group, including as compared with salamanders, counter to prior predictions for these sister taxa based on species richness alone^2^. Further, high diversification rates occur in clades using preformation such as some birds, teleosts and snakes as well as in clades with induction such as lizards and eutherian mammals^6^,^35^, together suggesting diversification rates are unrelated to PGC-specification mode in those vertebrates.

Among insects, the order Diptera (Supplementary Table 3) comprises a large diverse group of >240,000 species that specify germ cells using preformation^36^,^37^. However, its sister clades Lepidoptera and Coleoptera, with many taxa exhibiting induction^38^ also exhibit remarkable species diversity, with estimates of >174,000 (refs 39, 40) and 390,000 described species, respectively^41^. In fact, the Coleoptera, containing numerous induction species^38^ is the most speciose insect order^41^. Even within the family level of these insects, we find no consistent trends suggestive of higher species richness under preformation than under induction. As an example, the family Drosophilidae (preformation) contains about 4,000 species^42^, while other families of Diptera (for example, Nemestrinidae; also with preformation) contain as few as 300 species^43^. In turn, the Coleopteran family Tenebrionidae (containing the induction species *Tribolium castaneum*^44^,^45^ and *Tenebrio molitor*^46^,^47^) represents >20,000 species^48^, while the Lepidopteran family Bombycidae (containing *Bombyx mori*, also with the induction mode, as cited in Supplementary Table 4 (refs 49, 50, 51, 52, 53, 54) consists of 21 genera with just 150 species. Importantly, as noted by Wiegmann *et al*.^55^, the Diptera (preformation), Coleoptera (induction), Hymenoptera (*Apis* (preformation) and *Nasonia* (induction)), and the Lepidoptera (induction) are four superradiators in insects, and account for the majority of animal life on earth. Additional examples are provided in Supplementary Note 6. Most importantly, given that we observed no molecular evolutionary evidence of release of constraint, or rapid protein sequence divergence, under preformation (Figs 2, 3, 4) the underlying mechanism contributing towards enhanced diversification in clades with germ plasm^2^ is unlikely to exist in animals. Taken together, there is no current rationale to anticipate higher genome or species diversification under preformation across animals.

We propose that the fact that germ plasm has evolved convergently across animal lineages does not necessitate a general trend towards liberated constraint and rapid protein evolution, and rather likely results from other mechanisms. For instance, convergent evolution of a germ plasm-driven mechanism for specifying PGCs could result from advantageous mutations in a small subset of genes, or from gene expression changes^56^,^57^ involved in the acquisition of germ plasm^3^. An alternate theory that has been proposed to explain the convergent evolution of germ plasm (preformation), is that it is simply a side-effect, or spandrel^58^, of a heterochronic shift^59^ in body plan specification mechanisms generally, from late to early development^60^,^61^. Organisms displaying the preformation mode of PGC specification also tend to have much of their early axial patterning and body plan specification determined maternally, by asymmetric deposition of regional determinants within the oocyte during oogenesis and early embryogenesis^62^. Under this hypothesis, germ plasm would be simply one of many such maternally supplied determinants, ensuring that the germ line, as well as, for example, the dorsoventral and anteroposterior axes, were established before or immediately following fertilization, without requiring extensive zygotic genome activity or zygotic cell–cell signalling. Quantitative empirical tests of this hypothesis, beyond establishing the strength of the correlation between germ plasm and other body plan determinants that appears to hold at least for well-established model organisms^62^, may prove challenging. However, with our study we have sought to highlight the fact that as with all convergently derived traits, the mechanism of specification of the animal germ line may not itself be a direct target of selection, but rather an indirect consequence of selection for a distinct trait or mechanism.

While herein we found no evidence supporting the PGC-specification hypothesis, PGC-specification mode could affect other parameters related to molecular evolution, such as the evolutionary rates of a small number of genes, or sites within genes, involved in the mechanisms of preformation or induction. For example, evolution of germ plasm related genes such as *oskar*, *vasa*, *nanos*, *piwi*, *tudor*, *pie-1* and others might well differ from those shown to be instrumental to induction, such as BMP or Wnt signalling pathway members^63^,^64^,^65^,^66^. To test this, further studies should assess the molecular evolutionary dynamics of specific PGC genes and pathways using large-scale phylogenetic analysis across many species per genus with preformation and those with induction, allowing measurements of site-specific positive and negative selection^67^. Other molecular evolutionary parameters that PGC-specification mode might plausibly impact are mutation frequency in germ lines^68^,^69^. Future research should assess population-level frequencies of mutations to test for adaptive evolution and relaxed selection in specific PGC genes^70^,^71^. The rapid expansion of genome-wide sequence data sets in invertebrates^72^ will allow assessment of positive selection in genes involved in germ plasm formation using phylogenetic approaches that span a wide range of taxa in the future.

## Methods

### Data extraction

For each taxon under study, CDS sequences were either downloaded directly from a public database, or extracted from genomic data (Supplementary Tables 1 and 2). In organisms where genomic DNA was available as assembled scaffolds (Supplementary Table 2), the CDS regions were extracted using Augustus^73^ set at default parameters, and trained using a related species from the same genus with annotated genome data. To ensure accurate identification of CDS from scaffolds, open reading frames were verified using codons with ORF predictor^74^. For our analyses, we removed any CDS with unknown or ambiguous nucleotides, or with one or more internal stop codons.

### Orthology identification and measurements of dN/dS

For the identification of orthologues among species pairs listed in Table 1 and Fig. 1, we used BLASTX^75^ of the genome-wide CDS, where the match with the lowest *e*-value (and *e*<10^−6^) in reciprocal BLASTX searches was identified as the orthologue. For genes with more than one similar isoform (varying by an exon, or point mutations), this method yields the longest isoform per gene among taxa. Genes not having the same match in both reciprocal BLASTX searches were excluded from further analysis. Intergeneric identification of orthologues was also conducted by reciprocal BLASTX.

Alignments of gene sequence across species were conducted at the codon level using the program MUSCLE^76^. The dN and dS values were determined using the Nei–Gojobori method after exclusion of all gaps^77^. MUSCLE alignments and dN and dS were each determined using MEGA-CC^78^. All CDS per species pair (Table 1, Fig. 1) with dS>0 were retained for analysis of dN/dS. As it has been posited that ambiguous alignments from distant organisms, and sequencing errors due to low coverage, could inflate or alter molecular evolution parameters reported in the literature, including dN and dS^11^,^79^, we examined only closely related species with full CDS herein. Further, in the interest of prudency, we repeated our entire analyses in Figs 2, 3, 4 excluding all genes having dS values above the 90th percentile, which are most apt to exhibit segments of misalignment, imprecise orthology matches across taxa, and/or an abundance of sequence errors (each which can affect measures of molecular evolution parameters^79^, and obtained results nearly identical to those reported in each figure (data not shown)). This cutoff prevented exclusion of high dN genes unless its matching dS was also unusually elevated.

### Expression profiling

Expression levels of high dN/dS CDS across development in *Drosophila* were determined using modENCODE RNA-seq data in FlyBase (www.flybase.org)^27^. Expression levels for high dN/dS CDS sets across the ten analogous developmental stages in *Tribolium*, *Nasonia* and *Apis* were inferred^80^ from the orthologues from the relatively closely related insect *Drosophila* (Fig. 1). We propose that this is a reasonable inference since (a) the general developmental progression of these insects is quite similar^81^,^82^ and (b) the developmental gene expression profiles in *Drosophila* are highly conserved even with divergent invertebrates from non-Arthropod phyla^80^, and thus apt to be similar in such closely related insects.

### Identification of taxa for study

Phylogenetic independence among the invertebrates studied in Table 1; Supplementary Table 1; Fig. 1 was determined using phylogenies derived from large-scale sequence data^83^,^84^,^85^.

The animal genera under study in Table 1 and Fig. 1 were chosen based on a well-established mode of PGC specification, public availability of whole-genome DNA sequences for two species from a single genus at the commencement of our analyses (September to October 2014), and lack of saturation in dS. The taxa we identified matching these criteria, and having suitable data for another genus to allow comparison within the same phylum (one exception, contrast 7), were included in our analysis of dN/dS (Table 1). We note that while frogs (*Xenopus* (anurans)) versus salamanders (*Ambystoma* (urodeles)) comprised a primary contrast used by Evans *et al*.^6^, and represents a well-established case of preformation and induction respectively, we believe the available urodele sequence data sets are currently not suitable for large analyses representative of the genome, and are unsuitable for calculation within-genus dN/dS (Table 1, Fig. 1). This is because sequence data for salamanders (Ambystoma *mexicanum* and *A. tigrinum*) mainly comprise modest-sized expressed sequence tags data sets (ESTs) (∼20,000 ESTs and 1,700 other nucleotide sequences) for *A. tigrinum* (National Center for Biotechnology Information, NCBI; http://www.ncbi.nlm.nih.gov; search by taxon name) and a mix of genomic, RNA-seq and ESTs for *A. mexicanum* (Evans *et al*.^6^), which after assembly and orthology identification in two species (per genus) yields small partial gene sets for study. For instance, Evans *et al*.^6^ reported 6,679 and 2,078 CDS after assembly for each of *A. mexicanum* and *A. tigrinum*, respectively, many of which did not have a start or stop codon and thus were partial CDS (covering only part of the reading-frame) (http://www.nottingham.ac.uk/~plzloose/phyloinc). We found that only 523 partial CDS were available to study after orthology searches between the two salamander species using TBLASTX (cutoff, *e*<10^−6^; NCBI http://www.ncbi.nlm.nih.gov/BLAST/blast_program.shtml). Further, since the CDS list in each species is incomplete, the CDS identified as predicted orthologues between species are most likely to be best hits between CDS lists, rather than true orthologues (since many true orthlogous CDS are likely absent due to poor expression, or small sequence sample size). In addition, the contigs are inherently biased towards highly expressed genes from the specific tissues used to create the complementary DNA libraries that these EST or RNA-seq collections were derived from (for *A. tigrinum*, ESTs were from various tissues such as brain or pooled tissues, while for *A. mexicanum*, the transcriptome was generated from a combination of oocytes, embryos and ESTs from various tissues such as the tail and limb blastema (NCBI; http://www.ncbi.nlm.nih.gov^6^), and are not an unbiased sample of CDS in the genome.

While teleosts (preformation) and cartilaginous fish (induction) were also major systems studied in Evans *et al*.^6^, we consider that the within-genus data sets are too small to study here and claim that they are a representative sample of the genome. As an example, the salmon/trout (teleost) species *Oncorhynchus mykiss*, *O. nerka*, *O. tshawytscha* and *O. kisutch* had 5,745, 2,582 and 1,520 and 707 CDS/contigs (many not covering the complete CDS; http://www.nottingham.ac.uk/~plzloose/phyloinc) respectively, and thus no pairing between two of these species would yield sufficient orthologous CDS for analysis. Similar to the problem with the salamander data, these sequences would likely provide few true orthologues among the species in this genus (and rather best hits). Similarly, a paired within-genus contrast for the cartilaginous fish (Acipenseriformes) *Acipenser ruthenus* with *A. transmontanus* or *A. sinesis* using sequence data that were examined in that investigation^6^ was not feasible as the latter two taxa had only 281 and 152 partial CDS available, respectively. Thus, despite the fact that these groups are of interest because of the compelling evidence regarding their modes of PGC specification, the assessments of evolutionary rates across the genome for these genera cannot be robustly performed at the moment, but must await the availability of whole genomic DNA sequence data. Note that all citations to the number of CDS or contigs per species studied in Evans *et al*.^6^ were obtained by downloading the fasta files from http://www.nottingham.ac.uk/~plzloose/phyloinc.

### Data availability

The genomic sequences studied herein are all publicly available and their locations are provided in Supplementary Table 2. The data that support the findings of this study are available from the corresponding author on request.

## Additional information

**How to cite this article:** Whittle, C. A. & Extavour, C. G. Refuting the hypothesis that the acquisition of germ plasm accelerates animal evolution. *Nat. Commun.* 7:12637 doi: 10.1038/ncomms12637 (2016).

## Supplementary Material

Supplementary Information Supplementary Figures 1-3, Supplementary Table 1-7, Supplementary Notes 1-6 and Supplementary References

## Figures and Tables

**Table 1:** The 12 within-genus species pairs used to measure dN/dS and the pairs of between-genus contrasts.

**Paired between-genus contrasts**	Genus*	**Within-genus species pairs**	**PGC-specification mode**
1	*Drosophila*	*D. melanogaster* and *D. simulans*	Preformation
	*Tribolium*	*T. castaneum* and *T. freemani*	Induction
			
2	*Schistosoma*	*S. japonicum* and *S. haematobium*	Preformation
	*Echinococcus*	*E. granulosus* and *E. multilocularis*	Induction
			
3	*Nasonia*	*N. vitripennis* and *N. giraulti*	Preformation
	*Apis*	*A. florea* and *A. mellifera*	Induction
			
4	*Falco*	*F. cherrug* and *F. peregrinus*	Preformation
	*Alligator*	*A. mississippiensis* and *A. sinensis*	Induction
			
5	*Xenopus*	*X. laevis* and *X. tropicalis*	Preformation
	*Pan*	*P. troglodytes* and *P. paniscus*	Induction
			
*Supplemental contrasts*			
6	*Anopheles*	*A. darlingi* and *A. gambiae*	Preformation
	*Tribolium*	*T. castaneum* and *T. freemani*	Induction
7	*Pristionchus*	*P. pacificus* and *P. exspectatus*	Preformation
	*Echinococcus*	*E. granulosus* and *E. multilocularis*	Induction

PGC, primordial germ cell.

^*^All 12 within-genus species pairs are independent and thus comparable across genera. The independent genera have been grouped into five phylogenetically independent between-genus contrasts (1–5), as well as two supplemental non-independent contrasts (6 and 7). For citations of evidence for PGC mode see Supplementary Table 1. Note that *Tribolium* and *Echinococcus* were used in two paired between-genus contrasts, for a total of 12 genera under study.
