# Supplementary material for: Refuting the hypothesis that the acquisition of germ plasm accelerates animal evolution
Source: Nat Commun. 2016 Aug 31;7:12637. doi: 10.1038/ncomms12637 (PMC5013649; doi:10.1038/ncomms12637)
Supplement: Supplementary Information — Supplementary Figures 1-3, Supplementary Table 1-7, Supplementary Notes 1-6 and Supplementary References [file ncomms12637-s1.pdf]

Supplementary Information

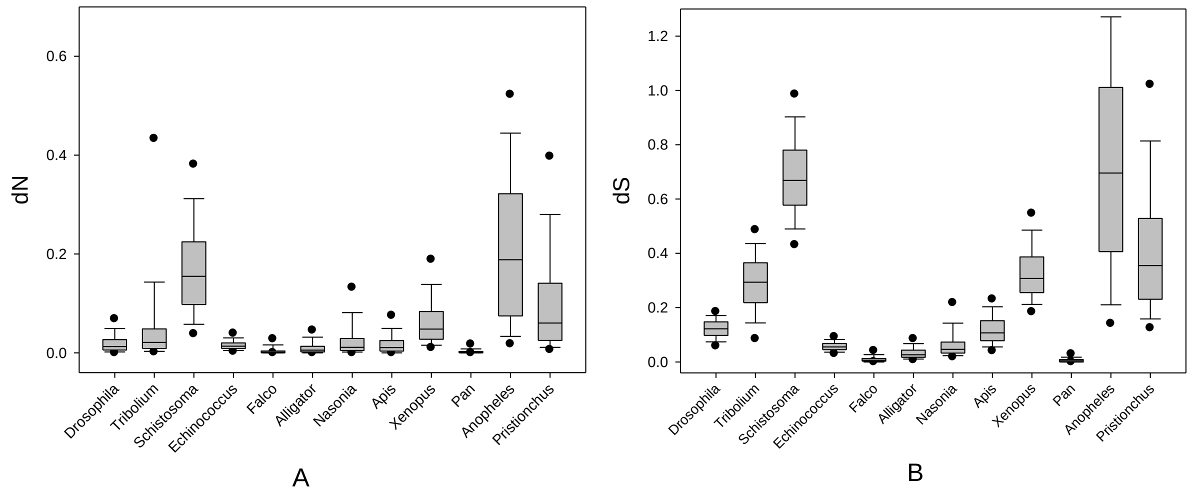

Supplementary Fig. 1. Box-whisker plots for the distribution of genome-wide (A) dN and (B) dS values for each of the twelve genera used in the dN/dS analysis.

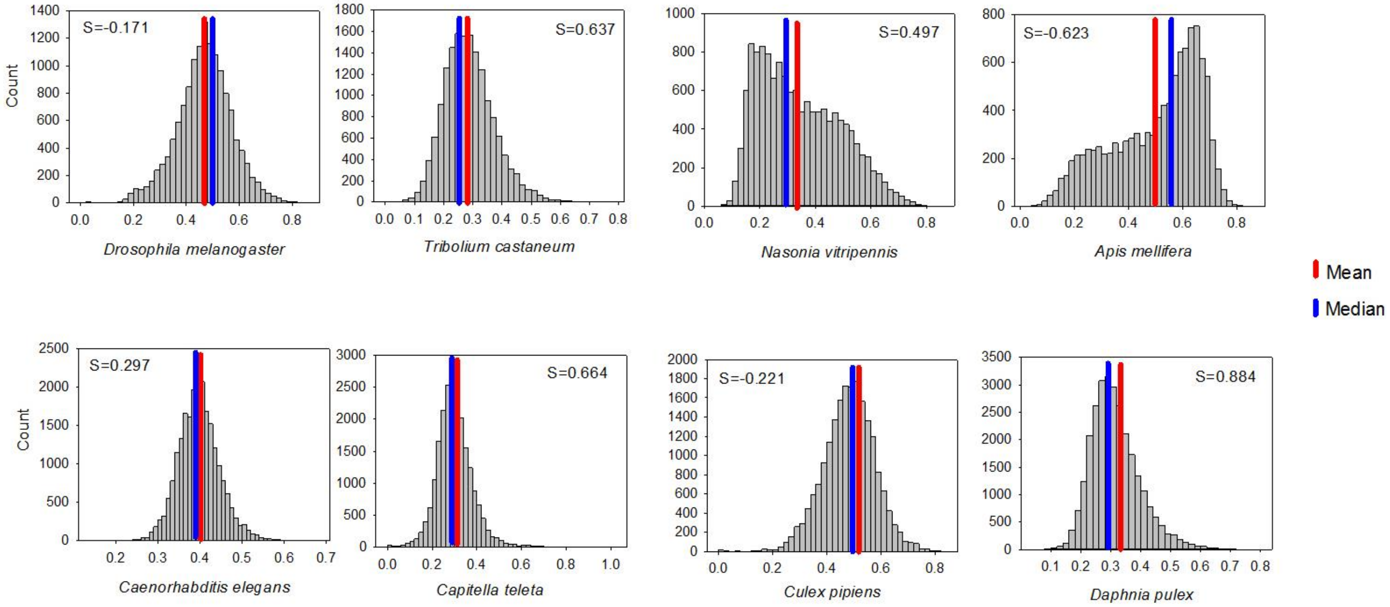

Supplementary Fig. 2. The frequency distribution of Fop per gene for each species under study.

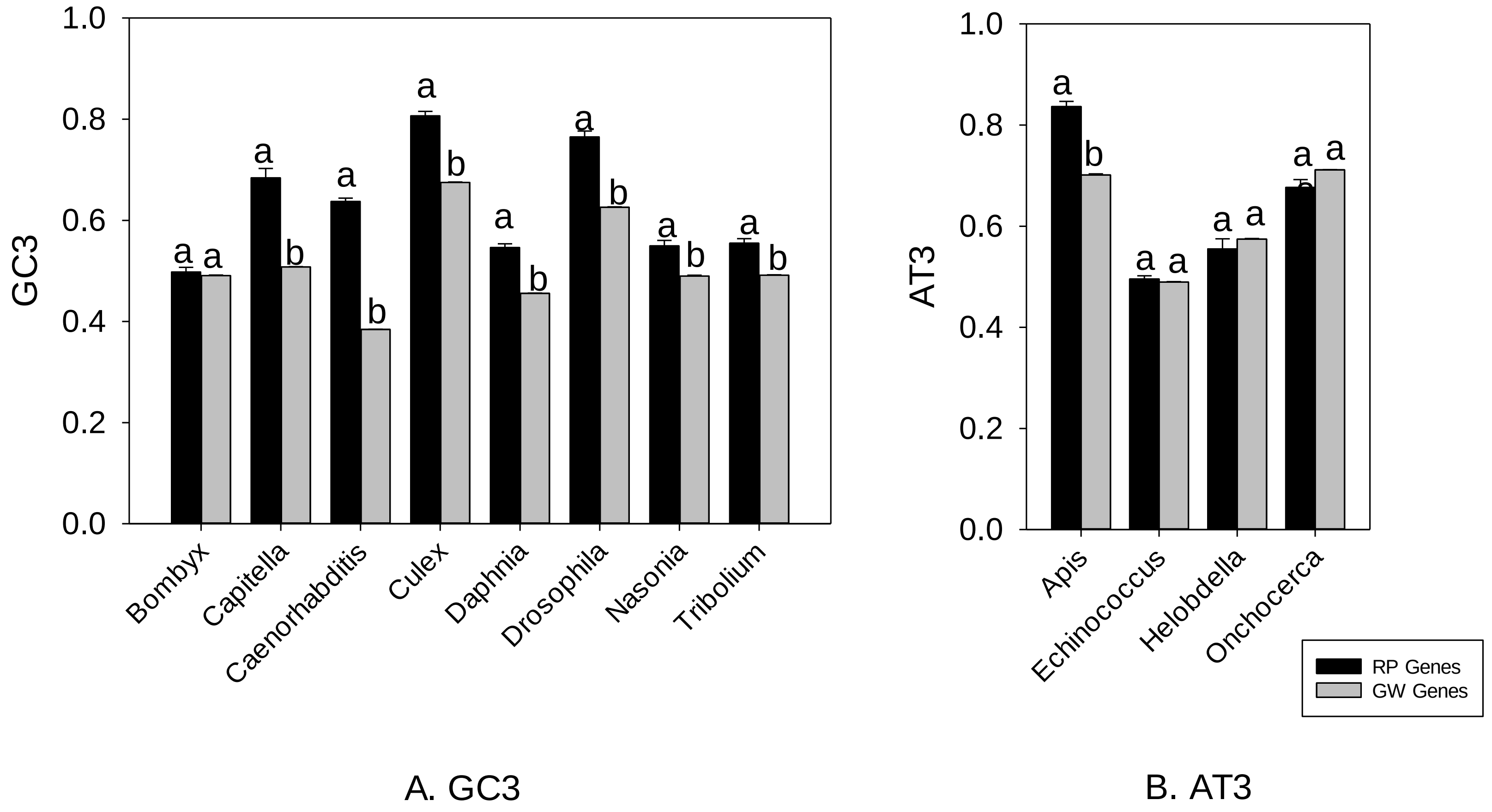

Supplementary Fig. 3. (A) The average GC3 content of ribosomal-protein genes (RP: black bars) and for all genes in the genome (GW: grey bars). Species listed are those wherein the putative optimal codons end in G or C (Supplementary Table 5). (B) The AT3 content for ribosomal protein genes (RP: black bars) and the genome-wide level (GW: grey bars) for species showing favoritism toward A- or T-ending codons (or no favoritism). Bars represent standard error. Species names are abbreviated using genus names.

Supplementary Table 1. The intrageneric species pairs and the intergeneric pairs used to compare dN/dS in the present study.

	Genera	Within-Genus Species Pair	PGC Specification Mode	Citation for PGC Specification Mode
Between Genus-Contrasts				
1	Drosophila	*D. melanogaster* and *D. simulans*	Preformation	[1-4]
	Tribolium	*T. castaneum* and *T. freemani*	Induction	[5,6]
2	Schistosoma	*S. japonicum* and *S. haematobium*	Preformation	[7-10]
	Echinococcus	*E. granulosus* and *E. multilocularis*	Induction	[11]
3	Nasonia	*N. vitripennis* and *N. giraulti*	Preformation	[12-14]
	Apis	*A. florea* and *A. mellifera*	Induction	[15-18]
4	Falco	*Falco cherrug* and *Falco peregrinus*	Preformation	[19]
	Alligator	*A. mississippiensis* and *A. sinensis*	Induction	[20]
5	Xenopus	*X. laevis* and *X. tropicalis*	Preformation	[21-26]
	Pan	*P. troglodytes* and *P. paniscus*	Induction	[27-29]
Supplemental Contrasts				
6	Anopheles	*A. darlingi* and *A. gambiae*	Preformation	[30]
	Tribolium	*T. castaneum* and *T. freeman*	Induction	See above

7	Pristionchus	*P. pacificus* and *P. exspectatus*	Preformation	[31,32]
	Echinococcus	*E. granulosus* and *E. multilocularis*	Induction	See above

Supplementary Table 2. The organisms examined in the present study and the location of their sequence datasets. Species were used in either dN/dS analysis and/or codon usage analysis. All datasets represent those versions available during the period of June to November 2014. Complete CDS were downloaded whenever possible, or were extracted from scaffolds. Note that genome Version Number is abbreviated as v.

**Taxon**	**Location of CDS or Scaffold Data**
dN/dS Analysis	
Alligator mississippiensis	**NCBI:** http://www.ncbi.nlm.nih.gov/bioproject/PRJNA221578 (Project ID PRJNA221578)
Alligator sinensis	**NCBI:** http://www.ncbi.nlm.nih.gov/bioproject/PRJNA221633 (Project ID PRJNA221633)
Anopheles darlingi	**Ensembl Genome:** http://metazoa.ensembl.org/Anopheles_darlingi/Info/Index (v. AdarC3.23)
Anopheles gambiae	**NCBI:** http://www.ncbi.nlm.nih.gov/bioproject/PRJNA163
Apis florea	**NCBI:** http://www.ncbi.nlm.nih.gov/refseq/ (Refseq v. 67, Organism *Apis florea*; Accessed Oct. 2014)
Apis mellifera	**Ensembl Genome:** http://metazoa.ensembl.org/Apis_mellifera/Info/Index (v. GCA_000002195.1.25)
Drosophila melanogaster	**FlyBase:** http://www.flybase.org (v. 5.57)
Drosophila simulans	**FlyBase:** http://www.flybase.org (v. r1.4)
Echinococcus granulosus	**Sanger:** http://www.sanger.ac.uk/resources/downloads/helminths/ (Accessed Oct. 2014)
Echinococcus multilocularis	**Sanger:** http://www.sanger.ac.uk/resources/downloads/helminths/ (Accessed Oct. 2014)
Falco cherrug	**NCBI:** http://www.ncbi.nlm.nih.gov/refseq/ (Refseq v. 67, Organism *Falco cherrug,* Accessed Oct. 2014*)*
Falco peregrinus	**NCBI:** http://www.ncbi.nlm.nih.gov/refseq/ (Refseq v. 67, Organism: *Falco peregrine;* Accessed Oct. 2014)
Nasonia giraulti	**NCBI:** http://www.ncbi.nlm.nih.gov/bioproject/PRJNA20223 (Project ID PRJNA2022; scaffolds)
Nasonia vitripennis	**Ensembl:**http://metazoa.ensembl.org/Nasonia_vitripennis/Info/Index **(**v. GCA_000002325.2.22)

Pan troglodytes	**Ensembl**: http://www.ensembl.org/Pan_troglodytes/Info/Index (v. CHIMP 2.1.4)
Pan paniscus	http://www.ncbi.nlm.nih.gov/refseq/ (Refseq v. 67, Organism: *Pan paniscus*; Accessed Oct. 2014)
Pristionchus pacificus	**Wormbase:** ftp://ftp.wormbase.org/pub/wormbase **(**v. WS246)
Pristionchus exspectatus	**Wormbase:** ftp://ftp.wormbase.org/pub/wormbase **(**v. WS246)
Schistosoma haematobium	**SchistoDB:** http://schistoDB.net/ (Accessed Oct. 2014)
Schistosoma japonicum	**SchistoDB:** http://schistoDB.net/ (Accessed Oct. 2014)
Tribolium castaneum	**Beetle Base:** http://beetlebase.org/ (http://metazoa.ensembl.org/Tribolium_castaneum)
Tribolium freeman	**Beetle Base:** http://beetlebase.org/ (Scaffold file name: tfre.scaffold0.fa; ftp://ftp.bioinformatics.ksu.edu/pub/BeetleBase/latest/)
Xenopus laevis	**Xenbase***:* http://xenbase.org (v. 6)
Xenopus tropicalis	**JGI:** http://genome.jgi-psf.org/Xentr4/Xentr4.info.html (v. 4)
Additional Taxa For Codon Usage Analysis	
Apis mellifera	See above
Bombyx mori	**Silkdb:** http://www.silkdb.org/silkdb/doc/download.html
Caenorhabditis elegans	**Wormbase:** http://www.wormbase.org/ (WBcel235.75)
Capitella teleta	**Joint Genome Institute (JGI):** http://genome.jgi-psf.org/Capca1/Capca1.download.ftp.html (v. 1)
Culex pipiens	**Broad Institute:** http://www.broadinstitute.org/annotation/genome/culex_pipiens.4/MultiDownloads.html (v. 4)
Daphnia pulex	**JGI:** http://genome.jgi-psf.org/Dappu1/Dappu1.download.ftp.html (v. 1)
Drosophila melanogaster	See above
Echinococcus granulosus	See above
Helobdella robusta	**Ensembl**: http://metazoa.ensembl.org/Helobdella_robusta/Info/Index (v. GCA_000326865.1)

Nasonia vitripennis	See above
Onchocerca volvulus	**Wormbase**: ftp://ftp.wormbase.org/pub/wormbase/releases/WS245/species/o_volvulus/PRJEB513/ **(**v. WS246)
Tribolium castaneum	See above

Supplementary Table 3. The taxa examined in the present study, their phylum, class, order and family, and the number of putative orthologs within genera. For dN/dS, two pairs of species were examined per genera. Genera with opposite PGC modes were grouped into five phylogenetically independent contrasts (numbered in leftmost column). The number of orthologous CDS was determined after reciprocal BLASTX and removal of all sequences with any ambiguous nucleotides or internal stop codons. The identified paired putative orthologs per genus were processed and analyzed as described in Methods and Supplementary Note 1.3. See Table 1 for citations for PGC specification mode for each genus.

	Genera	Species Pair per Genera	Phylum, Class, Order, Family	PGC Specification Mode	No. of Putative Orthologous CDS
Primary dN/dS Contrasts					
1	Drosophila	*D. melanogaster* and<br>*D. simulans*	Kingdom: Animalia<br>Phylum: Arthropoda<br>Subphylum (hexapods)<br>Class Insecta<br>Order Diptera<br>Family: Drosophilidae	Preformation	11,896
	Tribolium	*T. castaneum* and<br>*T. freemani*	Kingdom: Animalia<br>Phylum: Arthropoda<br>Subphylum: Hexapoda<br>Class: Insecta<br>Order: Coleoptera<br>Family: Tenebrionidae	Induction	5,656
2	Schistosoma	*S. japonicum* and<br>*S. haematobium*	Kingdom: Animalia<br>Phylum: Platyhelminthes<br>Class: Trematoda<br>Subclass: Digenea<br>Order: Strigeidida<br>Family: Schistosomatidae	Preformation	6,189

	Echinococcus	*E. granulosus* and *E. multilocularis*	Kingdom: Animalia<br>Phylum: Platyhelminthes<br>Class: Cestoda<br>Order: Cyclophyllidea<br>Family: Taeniidae	Induction	9,208
3	Nasonia	*N. vitripennis* and *N. giraulti*	Kingdom: Animalia<br>Phylum: Arthropoda<br>Class: Insecta.<br>Order: Hymenoptera<br>Family: Pteromalidae	Preformation	7,058
	Apis	*A. florea* and *A. mellifera*	Kingdom: Animalia<br>Phylum: Arthropoda<br>Class: Insecta<br>Order: Hymenoptera<br>Family: Apidae	Induction	6,869
4	Falco	*Falco cherrug* and *Falco peregrinus*	Kingdom: Animalia<br>Phylum: Chordata<br>Class: Aves<br>Order: Falconiformes<br>Family: Falconidae	Preformation	8,659
	Alligator	*A. mississippiensis* and *A. sinensis*	Kingdom: Animalia<br>Phylum: Chordata<br>Class: Reptilia<br>Superorder: Crocodylomorpha<br>Order: Crocodilia<br>Family: Alligatoridae	Induction	11,376

5	Xenopus	*X. laevis* and<br>*X. tropicalis*	Kingdom: Animalia<br>Phylum: Chordata<br>Class: Amphibia<br>Order: Anura<br>Family: Pipidae<br>Subfamily: Xenopodinae	Preformation	8,926
	Pan	*P. troglodytes* and<br>*P. paniscus*	Kingdom: Animalia<br>Phylum: Chordata<br>Class: Mammalia<br>Order: Primates<br>Family: Hominidae	Induction	10,479
Supplemental dN/dS Contrasts					
6	Anopheles	*A. darlingi* and<br>*A. gambiae*	Kingdom: Animalia<br>Phylum: Arthropoda<br>Subphylum (hexapods)<br>Class Insecta<br>Order Diptera<br>Family: Culicidae	Preformation	7,483
	Tribolium	*T. castaneum* and<br>*T. freeman*	See above	Induction	
7	Pristionchus	*P. pacificus* and *P.*<br>*exspectatus*	Kingdom: Animalia<br>Phylum: Nematoda<br>Class: Chromadorea<br>Order: Rhabditida<br>Family: Diplogastridae	Preformation	8,829

Echinococcus	*E. granulosus* and *E. multilocularis*	See above	Induction

Supplementary Table 4. The species studied for optimal codon usage and their PGC specification mode.

	Genus	Species	Phylum, Class, Order, Family	PGC Specification Mode	Citation for PGC Specification Mode
1	Apis	*Apis mellifera*	Phylum: Arthropoda<br>Class: Insecta.<br>Order: Hymenoptera.<br>Family: Apidae	Induction	[15-18]
2	Bombyx	*Bombyx mori*	Phylum: Arthropoda<br>Subphylym (hexapods)<br>Class Insecta<br>Order Lepidoptera<br>Family: Bombycidae	Induction	[33-39]
3	Caenorhabditis	*Caenorhabditis elegans*	Phylum: Nematoda<br>Class: Chromadorea<br>Order: Rhabditida<br>Family: Rhabditidae	Preformation	[40-42]
4	Capitella	*Capitella teleta*	Phylum: Annelida<br>Class: Polychaeta<br>Subclass: Scolecida<br>Family: Capitellidae	Induction	[43-45]
5	Culex	*Culex pipiens*	Phylum: Arthropoda<br>Subphylum (hexapods)<br>Class Insecta<br>Order Diptera<br>Flies: Culicidae	Preformation	[30,46]
6	Daphnia	*Daphnia pulex*	Phylum: Arthropoda<br>Subphylum: Crustacea<br>Class: Branchiopoda<br>Order: Cladocera<br>Family: Daphniidae	Preformation	[47]
7	Drosophila	*Drosophila melanogaster*	Phylum: Arthropoda<br>Subphylum (hexapods)<br>Class Insecta<br>Order Diptera<br>Family: Drosophilidae	Preformation	[1-4]

8	Echinococcus	*Echinococcus granulosus*	Phylum: Platyhelminthes<br>Class: Cestoda.<br>Order: Cyclophyllidea.<br>Family: Taeniidae	Induction	[11]
9	Helobdella	*Helobdella robusta*	Phylum: Annelida<br>Class: Clitellata<br>Subclass: Hirudinea<br>Order:Rhynchobdellida<br>Family:Glossiphoniidae	Induction	[48,49]
10	Nasonia	*Nasonia vitripennis*	Phylum: Arthropoda<br>Class: Insecta.<br>Order: Hymenoptera<br>Family: Pteromalidae	Preformation	[12-14]
11	Onchocerca	*Onchocerca volvulus*	Phylum: Nematoda<br>Class: Secernentea<br>Order: Spirurida<br>Family: Onchocercidae	Preformation	[32,50]
12	Tribolium	*Tribolium castaneum*	Phylum: Arthropoda<br>Subphylym (hexapods)<br>Class Insects<br>Order Coleoptera<br>Family: Tenebrionidae	Induction	[5,6]

Supplementary Table 5. The putative optimal codons per amino acid for the 12 taxa under study herein. Putative optimal codons were determined using ΔRSCU values from CDS sequences of genes with the highest versus the lowest 3% of ENC values (high versus low codon usage bias (CUB)). The preference for GC3 or AT3 codons is also shown. The putative optimal codon per amino acid is in bold for each taxon. P-values of t-tests among genes with high versus low ENC after correction for multiple tests are shown with asterisks: $*10^{-10}>P<0.05$; $**P\leq10^{-10}$. The "+" symbol indicates a gain in frequency of a codon in highly biased genes, while " –" indicates reduced level of the codon. Species names correspond to those presented in Supplementary Table 4 and have been abbreviated by genus name. Preformation has been abbreviated as P and induction as I. This table should be taken in conjunction with Supplementary Fig. 3 as described in Supplementary Note 1.4.

		Taxon											
**Optimal Codons**		**Apis**	**Bombyx**	**Capit.**	**Caeno.**	**Culex**	**Daphnia**	**Droso.**	**Echin.**	**Helob.**	**Nason.**	**Oncho.**	**Tribo.**
**Number per Taxon**		18	17	8	15	17	16	18	0	6	18	11	11
**GC3 or AT3 Biased**		AT	GC	GC	GC	GC	GC	GC	-	AT	GC	AT	GC
**PGC Mode**		I	I	I	P	P	P	P	I	I	P	P	I
						**Δ RSCU Values**							
**Amino Acid**	**Codon**												
Ala	GCT	+0.47**	-0.44**	-0.2*	+0.51**	-0.47**	+0.04	-0.37**	-0.08	+0.17	-0.72**	+0.1	-0.11
Ala	GCC	-0.77**	+0.3*	**+0.3***	**+0.66****	**+1.05****	**+0.4****	**+1.34****	+0.01	-0.38*	**+1.25****	-0.33*	**+0.41****
Ala	GCA	**+1.14****	-0.25*	-0.18*	-0.63**	-0.69**	-0.26*	-0.68**	-0.04	**+0.3***	-0.79**	**+0.46***	-0.27*
Ala	GCG	-0.94**	**+0.34***	-0.08	-0.6**	-0.12	-0.22*	-0.25*	-0.03	-0.19*	+0.27*	-0.35*	-0.05
Arg	CGT	+0.07	-0.36*	-0.04	+1.2**	+0.12	-0.01	+0.37*	+0.24	-0.13	-0.42*	-0.06	-0.25*
Arg	CGC	-0.52**	**+1.01****	**+0.34***	**+0.45***	**+1.03****	**+0.78****	**+2.08****	+0.01	-0.33*	**+1.24****	-0.28*	**+0.24***
Arg	CGA	-0.36*	-0.4**	-0.59**	-0.91**	-0.78**	-0.37*	-0.87**	-0.21	-0.33*	-0.66**	**+0.63***	-0.2*
Arg	CGG	-0.52**	+0.31*	-0.23*	-0.64**	+0.13	-0.22*	-0.33*	-0.22	-0.32**	+0.43**	-0.21	+0.05
Arg	AGA	**+2.13****	-0.26	-0.03	+0.24	-0.33*	-0.11	-0.82**	-0.07	**+1.19****	-0.94**	+0.27	-0.01
Arg	AGG	-0.83**	-0.33*	+0.12	-0.61**	-0.42**	-0.25*	-0.55**	+0.15	-0.19	+0.33*	-0.46*	+0
Asn	AAT	**+0.67****	-0.39**	-0.15*	-0.58**	-0.56**	-0.25**	-0.66**	-0.07	+0.07	-0.84**	**+0.28***	-0.24*

Asn	AAC	-0.69**	+0.31**	-0.01	+0.54**	+0.47**	+0.15*	+0.67**	-0.08	-0.11*	+0.79**	-0.28*	+0.13*
Asp	GAT	+0.65**	-0.43**	-0.17*	-0.25*	-0.45**	-0.21**	-0.39**	-0.08	+0.1	-0.8**	+0.11	-0.2*
Asp	GAC	-0.68**	+0.37**	+0.01	+0.09	+0.29**	+0.1*	+0.37**	-0.05	-0.19*	+0.77**	-0.22*	+0.08
Cys	TGT	+0.71**	-0.33**	-0.38**	-0.39**	-0.41**	-0.34**	-0.53**	-0.04	+0	-0.61**	+0.01	-0.27*
Cys	TGC	-0.78**	+0.26*	-0.14	+0.17*	+0.04	-0.05	+0.44**	-0.15	-0.3**	+0.57**	-0.18	-0.09
Gln	CAA	+0.49**	-0.34**	-0.37**	+0.12	-0.58**	-0.23**	-0.76**	-0.08	+0.1	-0.77**	+0.11	-0.1
Gln	CAG	-0.53**	+0.28*	+0.11	-0.22*	+0.48**	+0.12*	+0.73**	-0.1	-0.26**	+0.76**	-0.24*	+0
Glu	GAA	+0.68**	-0.41**	-0.26**	-0.35**	-0.49**	-0.16*	-0.75**	-0.1	+0.12*	-0.86**	+0.24*	-0.18*
Glu	GAG	-0.69**	+0.37**	+0.07	+0.26*	+0.38**	+0.11*	+0.71**	+0.02	-0.24**	+0.84**	-0.27*	+0.08
Gly	GGT	+0.39*	-0.39*	-0.11	-0.39**	-0.14	-0.16*	-0.23*	+0.04	+0.08	-0.66**	+0.33	-0.16
Gly	GGC	-0.79**	+0.54**	+0.28*	-0.63**	+0.26*	+0.31*	+1.05**	-0.09	-0.2*	+1.44**	-0.4*	+0.15
Gly	GGA	+0.81**	-0.29*	-0.17*	+1.5**	-0.18	-0.02	-0.37*	-0.12	+0.17	-0.6**	+0.33	+0.03
Gly	GGG	-0.51**	+0.07	-0.27*	-0.44**	-0.23*	-0.22*	-0.44**	-0.07	-0.31**	-0.15*	-0.24*	-0.13
His	CAT	+0.69**	-0.37**	-0.19*	-0.31**	-0.47**	-0.32**	-0.5**	-0.15	+0	-0.7**	+0.1	-0.18*
His	CAC	-0.74**	+0.28*	-0.09	+0.07	+0.24*	+0.12*	+0.5**	-0.04	-0.24*	+0.72**	-0.23*	+0.04
Ile	ATT	+0.41**	-0.43**	-0.3*	-0.44**	-0.57**	-0.24*	-0.5**	-0.01	+0.09	-0.82**	+0.29*	-0.21*
Ile	ATC	-0.86**	+0.56**	+0.3*	+0.82**	+0.83**	+0.36**	+1.19**	+0.02	-0.17*	+1.36**	-0.25*	+0.31*
Ile	ATA	+0.46**	-0.23*	-0.18*	-0.45**	-0.39**	-0.25**	-0.71**	-0.1	+0.01	-0.55**	-0.09	-0.24*
Leu	TTA	+2.7**	-0.37*	-0.1	-0.54**	-0.26*	-0.32**	-0.61**	-0.06	+0.51**	-0.78**	+0.86**	-0.19
Leu	TTG	-0.71**	-0.48**	-0.13	-0.4*	-0.63**	+0.3*	-0.67**	+0.06	+0.16	-0.93**	+0.26	+0.17
Leu	CTT	-0.05	-0.41**	-0.41**	+1.01**	-0.62**	-0.26*	-0.65**	-0.03	-0.12	-0.71**	-0.28*	-0.15
Leu	CTC	-0.77**	+0.45*	-0.11	+1.27**	-0.12	+0.24*	+0.1	+0.25	-0.33**	+1.82**	-0.2	+0.13
Leu	CTA	-0.21*	-0.29*	-0.24*	-0.56**	-0.44**	-0.3**	-0.58**	-0.22*	-0.02	-0.62**	-0.23	-0.34**
Leu	CTG	-0.94**	+1.08**	+0.83**	-0.76**	+2.08**	+0.27*	+2.42**	-0.02	-0.32*	+1.23**	-0.44*	+0.33*
Lys	AAA	+0.57**	-0.42**	-0.39**	-0.7**	-0.58**	-0.19*	-0.73**	-0.11	+0.12*	-0.87**	+0.2*	-0.17*
Lys	AAG	-0.57**	+0.32**	+0.24**	+0.65**	+0.52**	+0.1*	+0.73**	-0.02	-0.16*	+0.87**	-0.22*	+0.13*
Phe	TTT	+0.79**	-0.34**	-0.29**	-0.67**	-0.43**	-0.29**	-0.8**	-0.08	+0.11	-0.75**	+0.26*	-0.28*

Phe	TTC	-0.82**	**+0.27***	+0.01	**+0.59****	**+0.34****	**+0.14***	**+0.79****	-0.03	-0.27**	**+0.72****	-0.27*	**+0.13***
Pro	CCT	+0.28*	-0.37*	-0.43**	-0.58**	-0.45**	-0.18*	-0.39**	-0.02	-0.05	-0.69**	-0.26	-0.27*
Pro	CCC	-0.49**	**+0.28***	+0.19	-0.54**	+0.11	**+0.21***	**+1.17****	+0	-0.4**	**+1.03****	-0.14	**+0.33***
Pro	CCA	**+1.19****	-0.36*	-0.2*	**+1.83****	-0.62**	-0.1	-0.59**	+0.08	**+0.4***	-0.92**	+0.25	-0.2
Pro	CCG	-0.93**	+0.28*	+0.05	-0.8**	**+0.58****	-0.07	-0.2*	-0.18	-0.41**	+0.52**	-0.02	-0.09
Ser	TCT	+0.81**	-0.27*	-0.01	+0.44*	-0.4**	+0	-0.44**	+0.07	+0.12	-0.76**	-0.14	-0.26*
Ser	TCC	-0.72**	**+0.39***	+0.09	**+1.16****	**+0.58****	+0.08	**+1.09****	-0.05	-0.33**	+0.47**	-0.27*	**+0.29***
Ser	TCA	**+1.17****	-0.45*	-0.21*	-0.29*	-0.56**	-0.31*	-0.69**	+0.03	+0.19	-0.74**	**+0.83****	-0.26*
Ser	TCG	-1.01**	+0.39*	-0.04	-0.41**	+0.49*	+0	+0.26*	-0.03	-0.38**	+0.48**	-0.24	+0
Ser	AGT	+0.56**	-0.34*	-0.07	-0.71**	-0.51**	-0.14*	-0.69**	+0.01	+0.21	-0.71**	+0.1	+0
Ser	AGC	-0.87**	+0.15	+0.1	-0.21*	+0.19	**+0.33***	+0.5**	-0.05	+0.04	**+1.26****	-0.27*	+0.07
Thr	ACT	+0.55**	-0.33*	-0.14	-0.01	-0.53**	-0.17	-0.54**	+0.04	+0.1	-0.79**	+0	-0.21*
Thr	ACC	-0.81**	+0.25*	**+0.29***	**+1.13****	**+0.87****	**+0.41****	**+1.49****	+0.05	-0.28*	**+1.18****	-0.2*	**+0.34***
Thr	ACA	**+1.25****	-0.35*	-0.3*	-0.68**	-0.51**	-0.2*	-0.8**	-0.03	**+0.43****	-0.81**	**+0.33**	-0.28*
Thr	ACG	-1.02**	**+0.28***	-0.12	-0.54**	+0.02	-0.11*	-0.22*	-0.11	-0.35**	+0.38*	-0.25*	+0.06
Tyr	TAT	**+0.78****	-0.37**	-0.15*	-0.47**	-0.41**	-0.3**	-0.67**	-0.11	+0.14	-0.66**	**+0.21***	-0.21*
Tyr	TAC	-0.77**	**+0.28***	-0.16*	**+0.44****	**+0.3****	+0.11	**+0.66****	-0.12	-0.29*	**+0.67****	-0.2*	+0.1
Val	GTT	+0.75**	-0.43**	-0.35**	+0.11	-0.45**	-0.13*	-0.59**	-0.07	**+0.29***	-0.84**	+0.26	-0.19
Val	GTC	-0.71**	+0.04	**+0.23***	**+0.88****	**+0.65****	**+0.3***	+0.38**	-0.13	-0.38**	**+1.17****	-0.32*	-0.01
Val	GTA	**+0.87****	-0.3*	-0.16*	-0.47**	-0.49**	-0.23*	-0.6**	-0.05	+0.06	-0.64**	+0.23	-0.09
Val	GTG	-0.9**	**+0.64****	+0.07	-0.54**	+0.23*	-0.01	**+0.79****	+0.18	-0.1	+0.31*	-0.25	**+0.23***

Supplementary Table 6. The GC3 content of genes with the upper 3% codon usage bias (lowest ENC) and for the genome-wide CDS in the 13 taxa under study. The CDS were concatenated prior to calculation of GC3.

	Apis	Bombyx	Caeno.	Capit.	Culex	Daphnia	Droso.	Echin.	Helob.	Nason.	Oncho.	Tribo.
**GC3 of 3% Most-Biased Genes**	0.09	0.77	0.52	0.58	0.73	0.62	0.78	0.51	0.35	0.89	0.21	0.55
**GC3 of Genome-Wide CDS**	0.32	0.49	0.37	0.51	0.68	0.46	0.62	0.49	0.429	0.5	0.28	0.49

Supplementary Table 7. A sample of 121 known developmental genes used in our study. The FlyBase identification number, gene name and gene symbol are shown for each gene. The expression profiles and dN/dS values are shown in Fig. 4.

**FB ID**	**Gene Name**	**Gene Symbol**
FBgn0000014	*abdominal A*	*abd-A*
FBgn0000015	*Abdominal B*	*Abd-B*
FBgn0010379	*Akt1*	*Akt1*
FBgn0000097	*anterior open*	*aop*
FBgn0031458	*anterior pharynx defective 1*	*aph-1*
FBgn0262739	*Argonaute-1*	*AGO1*
FBgn0004569	*argos*	*aos*
FBgn0000117	*armadille*	*arm*
FBgn0000114	*arrest*	*aret*
FBgn0000119	*arrow*	*arr*
FBgn0024491	*Bicoid interacting protein 1*	*Bin1*
FBgn0000179	*bifid*	*bi*
FBgn0014135	*branchless*	*bnl*
FBgn0005592	*breathless*	*btl*
FBgn0261787	*brunelleschi*	*bru*
FBgn0004856	*Bx42*	*Bx42*
FBgn0000250	*cactus*	*cact*
FBgn0262975	*cap-n-collar*	*cnc*
FBgn0000251	*caudal*	*cad*
FBgn0036827	*CG6843*	*CG6843*
FBgn0013764	*Chip*	*Chi*
FBgn0000382	*corkscrew*	*csw*
FBgn0000339	*cornichon*	*cni*
FBgn0014143	*crocodile*	*croc*
FBgn0000394	*crossveinless*	*cv*

FBgn0004859	*cubitus interruptus*	*ci*
FBgn0000405	*Cyclin B*	*CycB*
FBgn0000490	*decapentaplegic*	*dpp*
FBgn0000439	*Deformed*	*Dfd*
FBgn0000524	*deltex*	*dx*
FBgn0000157	*Distal-less*	*Dll*
FBgn0010269	*Downstream of raf1*	*Dsor1*
FBgn0004638	*downstream of receptor kinase*	*drk*
FBgn0000576	*empty spiracles*	*ems*
FBgn0004875	*encore*	*enc*
FBgn0003731	*Epidermal growth factor receptor*	*Egfr*
FBgn0000611	*extradenticle*	*exd*
FBgn0001085	*frizzled*	*fz*
FBgn0001078	*ftz transcription factor 1*	*ftz-f1*
FBgn0001079	*fused*	*fu*
FBgn0001077	*fushi tarazu*	*ftz*
FBgn0250823	*gilgamesh*	*gish*
FBgn0024234	*glass bottom boat*	*gbb*
FBgn0001148	*gooseberry*	*gsb*
FBgn0264495	*grappa*	*gpp*
FBgn0001139	*groucho*	*gro*
FBgn0001137	*gurken*	*grk*
FBgn0004644	*hedgehog*	*hh*
FBgn0015805	*Histone deacetylase 1*	*HDAC1*
FBgn0263782	*HMG Coemzyme A reductase*	*Hmgcr*
FBgn0001235	*homothorax*	*hth*
FBgn0004864	*hopscotch*	*hop*
FBgn0261434	*huckebein*	*hkb*
FBgn0001180	*hunchback*	*hb*

FBgn0037657	*hyrax*	*hyx*
FBgn0001320	*knirps*	*kni*
FBgn0001319	*knot*	*kn*
FBgn0001325	*Kruppel*	*Kr*
FBgn0002522	*labial*	*lab*
FBgn0011278	*ladybird early*	*lbe*
FBgn0002552	*lines*	*lin*
FBgn0002736	*mago nashi*	*mago*
FBgn0011648	*Mothers against dpp*	*Mad*
FBgn0011656	*Myocyte enhancer factor 2*	*Mef2*
FBgn0038872	*Negative elongation factor A*	*Nelf-A*
FBgn0017430	*Negative elongation factor E*	*Nelf-E*
FBgn0261617	*nejire*	*nej*
FBgn0039234	*nicastrin*	*nct*
FBgn0004647	*Notch*	*N*
FBgn0004102	*ocelliless*	*oc*
FBgn0002985	*odd*	*odd skipped*
FBgn0003002	*odd paired*	*opa*
FBgn0025360	*Optix*	*Optix*
FBgn0261885	*osa*	*osa*
FBgn0020622	*Pi3K21B*	*Pi3K21B*
FBgn0003089	*pip*	*pipe*
FBgn0019947	*Presenilin*	*Psn*
FBgn0053198	*presenilin enhancer*	*pen-2*
FBgn0004595	*prospero*	*pros*
FBgn0000273	*Protein kinase, cAMP-dependent, catalytic subunit 1*	*Pka-C1*
FBgn0003165	*pumilio*	*pum*
FBgn0043900	*pygopus*	*pygo*
FBgn0033649	*pyramus*	*pyr*

FBgn0037364	*Rab23*	*Rab23*
FBgn0003079	*Raf oncogene*	*Raf*
FBgn0004390	*Ras GTPase activating protein 1*	*RasGAP1*
FBgn0003205	*Ras85D*	*Ras oncogene at 85D*
FBgn0024194	*rasp*	*rasp*
FBgn0004795	*retained*	*retn*
FBgn0004635	*rhomboid*	*rho*
FBgn0003300	*runt*	*run*
FBgn0003345	*scalloped*	*sd*
FBgn0003463	*short gastrulation*	*sog*
FBgn0027363	*Signal transducing adaptor molecule*	*Stam*
FBgn0016917	*Signal-stansducer and activator of transcription protein at 92E*	*Stat92E*
FBgn0004666	*single-minded*	*sim*
FBgn0024291	*Sirtuin 1*	*Sirt1*
FBgn0003430	*sloppy paired 1*	*slp1*
FBgn0003450	*snake*	*snk*
FBgn0001965	*Sons of sevenless*	*Sos*
FBgn0261648	*spalt major*	*salm*
FBgn0020767	*Sprouty-related protein with EVH-1 domain*	*Spred*
FBgn0263396	*squid*	*sqd*
FBgn0030869	*Suppressor of Cytokine signaling at 16D*	*Socs16D*
FBgn0041184	*Suppressor of Cytokine Signaling at 36E*	*Socs36E*
FBgn0033266	*Suppressor of Cytokine Signling at 44A*	*Socs44A*
FBgn0005355	*Suppressor of fused*	*Su(fu)*
FBgn0004837	*Suppressor of Hairless*	*Su(H)*
FBgn0039734	*Tace*	*Tace*
FBgn0033652	*thisbe*	*ths*
FBgn0262473	*Toll*	*Tl*

FBgn0003867	*torso-like*	*tsl*
FBgn0265974	*tout-velu*	*ttv*
FBgn0086356	*tumbleweed*	*tum*
FBgn0003900	*twist*	*twi*
FBgn0003944	*Ultrabithorax*	*Ubx*
FBgn0004003	*windbeutel*	*wbl*
FBgn0004360	*Wnt oncogene analog 2*	*Wnt2*
FBgn0036141	*wntless*	*wls*
FBgn0016078	*wunen*	*wun*
FBgn0041087	*wunen-2*	*wun-2*

Supplementary Notes

Supplementary Note 1 (related to Fig. 2a)

Mann-Whitney U-tests across whole genome dN/dS support no consistent trends with respect to preformation and induction. dN/dS tended toward significantly higher values for preformation genera in only two cases (Drosophila (preformation) versus Tribolium (induction) and Nasonia (preformation) versus Apis (induction)), but was significantly higher for induction genera than preformation genera in two other cases (Echinococcus (induction) versus Schistosoma (preformation), and Pan (induction) versus Xenopus (preformation); $P<10^{-15}$ for all contrasts), and showed no significant difference between Falco (preformation) and Alligator (induction) (P=0.13). In summary, multiple independent paired contrasts of genome-wide dN/dS distributions across metazoans do not support a trend of rapid gene evolution under preformation.

We report in Fig. 2a that the taxa Anopheles (preformation) and Pan (induction) had among the highest fraction of their CDS with dN/dS >0.5 (>29%), and >1 (>4%) of all genera under study. These trends indicate that highly similar dN/dS distributions can occur across organisms with opposite PGC modes. For Anopheles in particular, the unusually high fraction of genes with accelerated protein evolution could be explained by a number of life history traits that are independent of PGC specification mode, for example, its role as a vector in malaria transmission, which likely requires rapid adaption to the host and gene evolvability [51,52]. Pristionchus (preformation) exhibited a similar dN/dS profile to that observed in numerous other organisms with varying PGC specification modes, including Drosophila (preformation), Tribolium (induction), Schistosoma (preformation), Apis (induction) and Xenopus (preformation), again suggesting no link between PGC specification mode and the global rate of evolution of protein sequences. Pristionchus (preformation) also had fewer CDS with dN/dS>0.5 than Echinococcus. Collectively, the genome-wide profiles of dN/dS provide no evidence for a tendency towards rapid genome evolution in preformation organisms in invertebrates nor in vertebrates.

Supplementary Note 2 (related to Fig. 2b Falco versus Alligator)

A total of 58.1% of the 2,537 CDS exhibiting >1.5 differences between the vertebrates Falco (preformation) and Alligator (induction), had elevated dN/dS in the induction taxon rather than the preformation taxon. Nevertheless, the two Falco species under study (*F. cherrug* and *F. peregrines*) have been shown to exhibit rapid evolution of orthologs as compared to other birds such as chicken, turkey and zebra finch [53]. Thus, even closely related species with preformation, can exhibit relatively fast or slow gene evolution within a single class (Aves) [53,54]. Moreover, recent findings indicate that alligators exhibit very slow rates of sequence evolution per unit time, as compared to birds [55]. Indeed, after converting our dN and dS values to rates per unit time using divergence time of at least 23 (Paleogene period) and 2.1 Mya, respectively [53,56], we obtained a more than 8 fold lower substitution rate in alligators than birds for each parameter (MWU-test $P<10^{-15}$; note that using the upper limit of 66mya for the Paleogene period, yields a 2.8 fold lower rate in alligators than birds). This agrees with the notion that alligators have an exceptionally low mutation rate, in fact the lowest found among vertebrates to date [55]. Nevertheless, our data show that dN/dS distributions exhibit no notable differences among birds and alligators (Fig. 2) at broad scale, suggesting a comparable propensity for relaxed or positive selection under preformation and induction in these vertebrates. We do not exclude differences in these taxa for specific groups of genes (or for any of the taxon pairs studied), but the results suggest no broad effect observable across the genome with respect to PGC-specification mode.

It is worth noting that birds, which have extensive publicly available intergeneric genome data, have been shown to exhibit variable dN/dS among lineages [57], have lower dN/dS than mammals, (induction) for genes from many GO classes [57], and their mtDNA dN/dS has been shown to be typically lower than crocodiles, proposed to result from their endothermic nature [58]. None of these observations is consistent with PGC-specification mode being a major factor shaping protein evolution in this vertebrate taxon.

Supplementary Note 3 (Excluding a Role of Saturation, Divergence time, and Population Size on Results in Fig. 2)

We address three important factors that could be hypothesized to account for the patterns we observed in dN/dS in our paired contrasts. First, for the analyses in Figs. 2ab, we verified that genome-wide dN and dS were unsaturated for all interspecies contrasts within genera. The mean and median of dN and dS values were well below 1 for each genus (Supplementary Fig. 1). Nonetheless, any genes identified as substantial outliers (dS >3) between putative orthologs (Supplementary Table 3) were excluded from analysis. For further stringency, we repeated all our entire analyses (Figs. 2-4) excluding all those genes per genus (per species-pair) with dS values above the 90th percentile to avoid any potential effect of saturation (as well as avoiding putative misalignments or orthology mismatches, see Methods) and found nearly identical results for all of our figures (results not shown). Thus overall, our results from dN/dS analyses of genome-wide unsaturated and independent contrasts of preformation and induction genera (Figs. 2ab) suggest no consistent connection between PGC specification mode and molecular evolution. We note that the species pair for Falco and for Pan had the lowest divergence in dN or dS among all species pairs (Supplementary Fig. 1). For these species pairs, similar to all other species pairs, we presented all orthologs with dN≥0 and dS>0 in Fig. 2, noting dN=0 were most common in these taxa. The median dS for genes studied (dS>0) with dN =0 (Median Falco=0.006, Median Pan=0.006) closely matched the median across studied genes (Median $Falco_{All\ Genes}$=0.007, Median $Pan_{All\ Genes}$=0.007); suggesting the cases with a zero value for dN were the result of purifying selection, rather than to insufficient evolutionary time to accumulate detectable mutations.

Second, it has been suggested that dN/dS in bacteria may be elevated for more closely related than distantly related species pairs, due to a time lag in removal of slightly deleterious mutations [59]. Such a phenomenon cannot explain the present results in the eukaryotes studied here. For example, for Drosophila (preformation), the species examined (*D. melanogaster* and *D. simulans*) have a divergence time of about 1.2 Mya [60] whilst the Tribolium (induction) species (*T. castaneum* and *T. freemani*) diverged >11.6 mya [61]. The fact that we found only a very marginal proportion of genes with elevated dN/dS in Drosophila rather than Tribolium (Figs. 2ab), despite the potential for the shorter divergence time in the preformation genus to enhance dN/dS, strengthens our conclusions. Similarly, divergence times are lower for the two species of Nasonia (preformation) (~1 Mya, [62,63] than for Apis (preformation) (approximately Miocene, 5-25 Mya, [64]. Thus, if divergence time affected dN/dS, the marginally higher values observed in Nasonia would be an overestimate, again strengthening our conclusions. The two Falco (preformation) species (Table 1) have a shorter divergence time (~2.1mya; [53]) than those from Alligator (induction) (>23 mya) [56], but despite a short divergence time that could possibly increase dN/dS for the preformation taxon, we still observed higher values under induction (Fig. 2b). Finally, the divergence time of the two species of Pan is lower (<1.6mya [65]) than that of the Xenopus species (50mya, [66]), and divergence times are, to our knowledge, not established for Schistosoma (preformation) and Echinococcus (induction) species studied here. Thus, we cannot formally exclude the possibility that the tendency for lower dN/dS under preformation than induction in these two cases was partly due to shorter divergence times for species with induction (Fig. 2b). However, we suggest this is unlikely given the lack of an

effect observed in all the other contrasts. Collectively, these trends point toward the conclusion that our results cannot be explained by divergence time variation.

Third, small effective population sizes (Ne) can enhance dN, and thus dN/dS, mainly for the subset of genes in the genome with large negative selection coefficients, by allowing more frequent fixation of deleterious amino acids [67]. We consider the role of population size here, and do not exclude the possibility that Ne had an effect on dN/dS. Rather, we argue that Ne could not explain our results. For instance, in the contrasts that opposed the preformation/induction theory (i.e., exhibited similar dN/dS under preformation and induction, or had higher dN/dS under induction), namely Drosophila (preformation) versus Tribolium (induction), Schistosoma (preformation) versus Echinococcus (induction), Pristionchus (preformation) versus Echinococcus (induction), Falco (preformation) versus Alligator (induction), and Xenopus (preformation) versus Pan (induction), the induction taxon could have had a history of smaller Ne or experienced more bottlenecks over its evolutionary history, leading to elevated dN/dS values for the induction taxon. However, this appears unlikely to have occurred for all five independent contrasts, and particularly for the insects Drosophila (preformation) and Tribolium (induction), and for the two contrasts involving Schistosoma (preformation), Echinococcus (induction) and Pristionchus (preformation) which all represent short-lived free-living or parasitic worms. Ne could have an effect for the comparison of Falco (preformation) versus Alligator (induction), where smaller populations or more bottlenecks may have occurred in the evolutionary history of the latter taxon (but this remains debatable [53,68]). Population size could also play a role in Xenopus (preformation) versus Pan (induction), wherein the latter taxon has a longer generation time (15 years, Stone et al. 2010; and is four months to two years in Xenopus, http://www.xenbase.org), which typically corresponds to a smaller population size [65,67,69]. However, even if Ne were smaller for the induction taxon in these two latter cases, if preformation is indeed the predominant factor accelerating protein evolution and liberating selective constraint, as concluded by Evans et al. [70], then it would be expected to counteract any effect of a small-Ne in the compared induction species; thus closing any gap in dN/dS values among preformation and induction or even yielding higher dN/dS under preformation. Taken together, we conclude that our findings are unlikely to be explained by population size, and that preformation does not accelerate dN/dS in the animals studied herein

<u>*Supplementary Note 4 (Frequency of Optimal Codons and PGC Mode)*</u>

As a complementary test to dN/dS, we studied the frequency of optimal codons (Fop) and report that this parameter is also uncorrelated to PGC specification mode. Optimal codons may not be present in every organism, but have been reported a wide range of animal systems, including Drosophila, Caenorhabditis, and Tribolium [71-73]. Analysis of optimal codon usage has been employed in Drosophila and other eukaryotes to detect rapidly evolving proteins [74-76], as proteins that are evolving rapidly appear to have low Fop [74-78]. The explanation for this relationship is twofold. First, purifying selection often affects proteins and codon usage similarly [77,79,80]. Thus, relaxed purifying selection on proteins may be detected as reduced Fop [77,80]. Second, positive selection on a protein sequence can reduce Fop due to selective sweeps, leading to fixation of non-optimal codons at linked gene sites[81-83]. Under the hypothesis of liberation of selective constraint on proteins from preformation species proposed by Evans et al. [70], which presumably includes relaxed selection and/or positive selection, we would expect to detect losses of optimal codons in organisms with preformation.

To test whether Fop is connected to PGC mode, we first needed to verify, or in some cases identify, the list of optimal codons for each taxon under study (see below "Identification of Optimal Codons" in Section 1.4). For this, we examined whole genome-CDS for twelve taxa that have publicly available large-scale DNA sequence datasets and a known mode of PGC formation (Supplementary Table 4). Within this species list, we included *D. melanogaster*, *T. castaneum*, *Nasonia vitripennis* and *Apis mellifera* as controls to compare to our

dN/dS findings, and eight additional species listed in Supplementary Table 4. In summary, we found optimal codons for the four aforementioned taxa as well as for the species *C. elegans*, *C. teleta*, *Culex pipiens*, and *Daphnia pulex* (for further details, see below “Identification of Optimal Codons”). Most of these species had putative optimal codons ending in GC3, but *A. mellifera* had AT3 putative optimal codons (Supplementary Table 5, also verified with ribosomal protein gene analysis, see “Identification of Optimal Codons”). Four of the twelve species studied had inconclusive or had no evidence of optimal codons. As species without optimal codons are not informative with regard to selection relative to PGC specification mode, these species were not included in subsequent analyses.

Using the optimal codon list for each of eight taxa, we studied the frequency distributions of gene Fop values across the genome (Supplementary Fig. 2). If an increased rate of protein sequence evolution arises due to relaxed and/or positive selection after an evolutionary transition to the preformation mode of PGC formation, then one would predict that a major portion of gene sequences should exhibit lowered Fop relative to the genome-wide Fop in such taxa [74,76]. Instead, we found that for all eight species under study, regardless of PGC specification mode, Fop appeared approximately normally distributed. This distribution profile is consistent with patterns previously observed for Fop (GC3) in Drosophila [84]. Nevertheless, for each species, Fop exhibited mild skewing, with mildness defined as $0<S<1$ for positive skewing, or $0>S>-1$ for negative skewing [85] (P-value of Kolmogorov-Smirnov test (K-S) of normality <0.05 for all species). While the absolute value of skewness (S) was <1 for each species, no severe cases of skewness ($S>2$) were observed.

The Fop distribution varied mildly among taxa. For instance, in our control species (those in which we had both dN/dS and Fop data) *D. melanogaster* (preformation) and *T. castaneum* (induction) very weak skewing was observed in each taxon (Supplementary Fig. 2), and agrees with the absent/very mild genome-wide differences detected between these taxa in dN/dS (Figs. 2ab). For *N. vitripennis* we found an abundance of low Fop values that clustered below the average (Supplementary Fig. 2), whilst *A. mellifera* showed on opposite clustering toward high Fop values. This is also consistent with the dN/dS analysis, which showed that a marginally greater portion of gene sequences had elevated dN/dS in *N. vitripennis* relative to *A. mellifera* (Fig. 2). Together, these results indicate that Fop reflects the patterns of genome-wide protein evolution as revealed by dN/dS analysis in these taxa [74,77,78]. Thus, we used Fop as a proxy for protein evolution in the remaining four organisms, which are described in the main text for Supplementary Fig. 2.

Identification of Optimal Codons (used to calculate Fop above)

We confirmed, or identified, optimal codon lists for twelve animal species in our study. Taxa and their PGC mode are listed in Supplementary Table 4, and include species of Drosophila. Tribolium, Nasonia, Apis, Bombyx, Capitella, Caenorhabditis, Culex, Daphnia, Echinococcus, Helobdella and Onchocerca. Putative optimal codons can be identified by asking which synonymous codons increase in frequency per amino acid as genes become more biased in codon usage [72], followed by verification of their abundance in highly expressed genes, such as ribosomal protein genes [86]. The effective number of codons (ENC) provides measure of the degree of codon usage bias irrespective of the type of bias (e.g., AT3 or GC3). When codons are all used at similar levels, the ENC has a high value (up to 61) whilst a greater bias results in a low ENC (as low as 20) [72,87]. Accordingly, to identify optimal codons in each taxon, we studied codon usage in the CDS with the highest 3% lowest ENC values versus those with the lowest 3% highest values. For each gene per gene set, we determined the relative synonymous codon usage (RSCU). A higher RSCU value for a codon in a synonymous codon family (amino acid) denotes increased usage [72,88]. Codons with biased usage were identified as those with the greatest change in RSCU among highly biased and low biased genes ($\Delta RSCU = RSCU_{High\ ENC} - RSCU_{Low\ ENC}$) [60,71,89] using t-tests corrected for multiple contrasts (Supplementary Table 5). As a second step, to confirm the

optimal codons were associated with gene expression, rather than mutational pressure, we examined ribosomal protein genes (RPGs), which are typically among the highest expressed and most conserved genes in most organisms [86,90]. In particular, we assessed whether codon usage in the highly expressed RPGs supported a role of selection in the optimal codons identified per organism [86].

Using *Drosophila melanogaster* and *Caenorhabditis elegans* wherein optimal codons have been identified a priori [60,71], we confirmed the effectiveness of the above approach to find optimal codons. For *D. melanogaster* and *C. elegans*, our results showed a strong preference for GC-ending codons (GC3): 100% of the optimal codons end in G or C (Supplementary Table 5). Further, the optimal codon list for *D. melanogaster* matches precisely that previously reported for this taxon (18 of 18 optimal codons) [60,71,72] For *C. elegans* we identified 15 of 18 the optimal codons previously reported for this taxon. Excluding our strict correction for multiple comparisons, an additional two optimal codons were identified for this taxon ($P<0.05$), which correspond to the same codons previously shown to exhibit a weak signal as optimal codons [60]. Thus, 17 of 18 optimal codons in this taxon match those previously reported using gene expression analyses [60]. Our RPG analyses also support the identity of optimal codons. For instance, for *D. melanogaster* (N_{RPGs}=87) and *C. elegans* (N_{RPGs}=82), GC3 content was statistically significantly higher in the RPGs than the genome-wide average (Supplementary Fig. 3). As optimal codons end in GC3 in these taxa, this suggests that selection is shaping their codon usage.

As codon usage studies from invertebrates other than *D. melanogaster* and *C. elegans* are less common, or absent, we determined the optimal codon list for the remaining species herein using the above approach. We report that optimal codon usage was evident within the Diptera, wherein ΔRSCU revealed that *Culex pipiens* (and *D. melanogaster*) each have a preference for GC3 optimal codons across synonymous codon families (Supplementary Table 5). Further, GC3 was statistically significantly higher for RPGs than for the genome-wide CDS (Supplementary Fig. 3), suggesting that the optimization of codon usage is shaped by expression-related selection in these organisms.

In the Hymenoptera, *Apis mellifera* and *Nasonia vitripennis* showed signals of having optimal codons (Supplementary Table 5). In *A. mellifera*, ΔRSCU indicated that the favored codons ended in A or T (AT3), and the association with expression was confirmed using RPGs (Supplementary Fig. 3). This differs from a recent report suggesting primarily GC3 optimal codons in this taxon (Carlini and Makowski 2015). However, as acknowledged in that assessment, *A. mellifera* showed a weak signature of optimal (or preferred as named therein) codon usage, lower than all other species studied, and the analysis of optimal codons did not include expression data. Hence, since we observed clear signals of AT3 optimal codons using ribosomal protein genes (as a measure of high expression) (Supplementary Fig. 3), we used our current optimal codon list for analysis. Nonetheless, future large-scale transcriptome datasets will confirm the definitive optimal codon list in this taxon . In *N. vitripennis*, putative optimal codons ended in G or C (GC3) (Supplementary Table 5, Supplementary Fig. 3); this agrees with a recent report for *N. vitripennis* [91]. We found that while *N. vitripennis* has substantial AT3 levels in CDS regions (50%, Supplementary Table 6), its optimal codons in highly biased genes are comprised of GC3 codons (Supplementary Table 5, Supplementary Fig. 3). In fact, for *N. vitripennis*, GC3 was 78% higher in the highly biased gene set (3% lowest ENC) than the genome-wide CDS (Supplementary Table 6), representing the strongest signal for the optimal codons among the organisms under study. This phenomenon parallels trends observed in Caenorhabditis where the genome-wide CDS has been reported to be AT3 rich [92], as observed here (AT3=0.63, Supplementary Table 6), but the optimal codons typically exhibit GC3 (Supplementary Table 5; also see [60,71]).

For the taxon *Bombyx mori* (Lepidoptera), we found evidence of biased codon usage, but the codon profiles appeared unlikely to be driven by selection. Specifically, ΔRSCU revealed preferential usage of GC3

codons for 17 of the 18 amino acids with synonymous codons in *B. mori* (Supplementary Table 5). However, the RPGs showed similar levels of GC3 as those observed in the genome-wide CDS (Supplementary Fig. 3), implying that codons with elevated ΔRSCU were common in these highly expressed genes. One possible explanation for this result is that RPGs exhibit uncharacteristically lowered expression in this taxon. To assess this possibility, we assembled a database using all *B. mori* ESTs available at NCBI, representing the testis, hemocyte, malphigian tubule, midgut or ovary tissues. We then compared the expression level of the RPG's and the 3% most biased genes for *B. mori*. Using the number of EST hits per gene as a measure of gene expression level [71,89], we found that the RPGs were highly expressed, and even had higher expression levels than the average for the 3% most biased genes (Average ESTs per 1,000 per gene= 1.62 and 0.37 respectively; t-test preformation value=$6.2X10^{-6}$). In contrast, the ribosomal proteins genes exhibited relatively low bias in codon usage, with an average ENC=52.4 (±0.68). In sum, we conclude that whilst selection might play some role in *B. mori* codon usage [93], no clear signal was evident herein, suggesting that other factors, such as mutational pressure, play a significant role in this particular taxon. This is consistent with recent reports for codon usage this taxon [94].

The taxon *Daphnia pulex* showed bias towards for GC3 codons (Supplementary Table 5). For *D. pulex*, the GC3 content of RPGs was greater than the genome-wide average, consistent with a role of expression-related selection in this taxon (Supplementary Fig. 3). Some taxa had moderate numbers of amino acids with an optimal codon including *Tribolium castaneum* (Arthropoda), and *Capitella teleta* (Annelida). For *T. castaneum* and *C. teleta* ΔRSCU showed a preference for GC3 in 11 and 8 amino acids, respectively. Further, GC3 was statistically significantly higher for RPGs than the genome-wide CDS in each taxon, indicating that these are indeed likely optimal codons shaped by selection (Supplementary Fig. 3). In *T. castaneum* a recent study assigning optimal codons as those with the strongest correlation values to expression, suggested favored codons end in GC, agreeing with our results, but suggested that preferences were found for 16 of 18 amino acids [73]. However, the effect was weak for some of the codons [86]. Nonetheless, due to the high stringency herein, we consider our putative optimal codon lists conservative.

Among the remaining organisms, *Helobdella robusta*, *Echinococcus granulosus*, and *Onchocerca volvulus* showed no evidence of selection mediated optimal codon usage. Although *H. robusta* (Annelida) showed six codons with preferential usage of AT3 (Supplementary Table 5), no difference was detected among RPGs and genome-wide AT3 (Supplementary Fig. 3), suggesting that this mild bias is not driven by selection. For *O. volvulus*, which favored AT3 codons, the AT3 of RPGs was showed no difference or was lower, respectively, than for the genome-wide AT3, inconsistent with the presence of optimal codons (Supplementary Fig. 3). The taxon *E. granulosus* (Platyhelminthes) was the only organism with no evidence of biased codon usage using ΔRSCU (Supplementary Table 5). Taken together, it is evident that RPGs suggest a role of selection in shaping optimal codon usage for eight of the twelve species studied, including *A. mellifera*, *C. elegans*, *C. pipiens*, *C. teleta*, *D. melanogaster*, *D. pulex*, *N. vitripennis*, and *T. castaneum*, with no or inconclusive signals of optimal codons for *E. granulosus*, *H. robusta*, *B. mori* and *O. volvulus*. Further studies using genome-wide expression will be needed to include/exclude optimal codons in those organisms. As species without signals of optimal codons are uninformative with regard to selection, we studied optional codon usage in the eight species with evidence of optimal codons, in order to evaluate whether PGC mode influences molecular evolution.

We note that for our four "control" species, *D. melanogaster*, *T. castaneum*, *Nasonia vitripennis* and *Apis mellifera* (used as controls to discern a relationship between dN/dS and Fop), we found that after binning of dN/dS into the four classes used in Fig. 2a ($dN/dS<0.5$, $0.5\leq dN/dS<0.75$, $0.75\leq dN/dS<1$, and $dN/dS\geq1$), there was an inverse correlation between dN/dS and Fop for *D. melanogaster*, (Spearman R=-1,P=0.017), *A.*

mellifera (R= -0.9 P<0.047), *N. vitripennis* (R=-1, P=0.017), and *T. castaneum* (R=-0.299, P=0.68), similar to trends suggested in other organisms [74,77,78]. In *T. castaneum*, whilst this correlation did have a negative R value, it was not statistically significant, perhaps because this taxon had fewer optimal codons than other species, making Fop values less strong than other species (Supplementary Table 5).

Supplementary Note 5 (dN/dS and Developmental Stage)

We note that whilst the percentage of high dN/dS CDS expressed at each developmental stage is the same between Drosophila and Tribolium, the absolute number of CDS with high dN/dS is slightly higher for Drosophila across all developmental stages, simply because the Drosophila-Tribolium contrast was one of two (among our five contrasts; the second such contrast was Nasonia-Apis) that had a marginally higher number of high dN/dS in the preformation taxon (MWU-tests $P<10^{-15}$, see Results for Fig. 2ab).

Supplementary Note 6.(Additional Examples of Speciation Under Preformation and Induction)

Among the two Platyhelminthes taxa studied here (Fig. 1), the genus Schistosoma (preformation) has 21 recognized species [95] and Echinococcus (induction) has nine species [96], thereby suggesting very low genus-level species richness under both PGC modes. The Annelida, a group that originated more than 516 Mya, is a highly diverse phylum with a minimum predicted 26,000 species [97]. The two divergent Annelid species examined here (Capitella and Helobdella, Fig. 1) both exhibit induction mode (Supplementary Table 4), suggesting that this mode of PGC formation (in at least some lineages) did not impede its high radiations. Other invertebrates also suggest PGC mode is unrelated to radiation across protostomes. For example, the Daphniidae (containing Daphnia, preformation [47,98,99]) have just 121 described species [100], while Aphididae (containing a number of preformation species including *Acyrthosiphon pisum* [101-104]) has approximately 4,300 [105]. Together, this suggests the preformation mode can be linked to low or high levels of radiation, based solely on family level species diversity. Collectively, these examples indicate that preformation and induction modes are uncorrelated to species radiations in invertebrates.

Supplementary References

1 Huettner, A. F. The origin of the germ cells in *Drosophila melanogaster*. *J. Morphol.* **2**, 385-422 (1923).

2 Poulson, D. F. Diagram of cell lineage in the embryo of *D. melanogaster*. *The Biology of Drosophila*, 243 (from 168-274) (1950).

3 Illmensee, K. & Mahowald, A. P. Transplantation of Posterior Polar Plasm in *Drosophila*. Induction of Germ Cells at the Anterior Pole of the Egg. *Proc. Natl. Acad. Sci. USA* **4**, 1016-1020 (1974).

4 Underwood, E. M., Caulton, J. H., Allis, C. D. & Mahowald, A. P. Developmental Fate of Pole Cells in *Drosophila melanogaster*. *Dev. Biol.*, 303-314 (1980).

5 Schroder, R. *vasa* mRNA accumulates at the posterior pole during blastoderm formation in the flour beetle *Tribolium castaneum*. *Dev. Genes Evol.* **216**, 277-283 (2006).

6 Handel, K., Grünfeld, C. G., Roth, S. & Sander, K. *Tribolium* embryogenesis: a SEM study of cell shapes and movements from blastoderm to serosal closure. *Dev. Genes Evol.*, 167-179 (2000).

7 Bednarz, S. The developmental cycle of the germ cells in several representatives of Trematoda (Digenera). *Zool. Pol.* **23**, 279-326 (1973).

8 Bednarz, S. The developmental cycle of germ cells in *Fasciola hepatica* L. 1758 (Trematoda, Digenera). *Zool. Pol.* **12**, 439-466 (1962).

9 Cort, W. W. The germ cell cycle in digenetic trematodes. *Quart. J. Microscop. Sci.* **19**, 275-284 (1944).

10 van der Woude, A. The germ cell cycle of *Megalodiscus temperatus* (Stafford, 1905) Harwood 1932 (Paramphistomidae: Trematoda). *Amer. Midl. Nat.* **51**, 172-202 (1954).

11 Gustafsson, M. K. S. Studies on cytodifferentiation in the neck region of Diphyllobothrium dendriticum Nitzeh 1824 (Cestoda, Pseudophyllidea). *Parasitenk* **50**, 323-329 (1976).

12 Bull, A. L. Stages of living embryos in the jewel wasp *Mormoniella (Nasonia) vitripennis* (Walker) (Hymenoptera: Pteromalidae). *International Journal of Insect Morphology and Embryology* **1**, 1-23 (1982).

13 Lynch, J. A. & Desplan, C. Novel modes of localization and function of *nanos* in the wasp *Nasonia*. *Development* **137**, 3813-3821, doi:10.1242/dev.054213 (2010).

14 Lynch, J. A. *et al.* The Phylogenetic Origin of *oskar* Coincided with the Origin of Maternally Provisioned Germ Plasm and Pole Cells at the Base of the Holometabola. *PLoS Genetics* **7**, e1002029, doi:10.1371/journal.pgen.1002029 (2011).

15 Bütschli, O. Zur Entwicklungsgeschichte der Biene. *Z. Wiss. Zool.* **20**, 519-564 (1870).

16 Fleig, R. & Sander, K. Blastoderm development in honey bee embryogenesis as seen in the scanning electron microscope. *International Journal of Invertebrate Reproduction and Development* **8**, 279-286 (1985).

17 Fleig, R. & Sander, K. Embryogenesis of the Honeybee *Apis mellifera* L (Hymenoptera, Apidae) - an SEM Study. *International Journal of Insect Morphology and Embryology* **15**, 449-462 (1986).

18 Nelson, J. A. *The embryology of the honey bee.*, (Princeton University Press, 1915).

19 Tsunekawa, N., Naito, M., Sakai, Y., Nishida, T. & Noce, T. Isolation of chicken *vasa* homolog gene and tracing the origin of primordial germ cells. *Development* **127**, 2741-2750 (2000).

20 Ferguson, M. W. J. in *Biology of the Reptilia* Vol. 14 (eds Carl Gans, Frank S. Billet, & Paul F. A. Maderson) 329-491 (Wiley & Sons, 1985).

21 Buehr, M. & Blackler, A. W. Sterility and partial sterility in the South African clawed toad following the pricking of the egg. *J. Embryol. Exp. Morphol.* **23**, 375-384 (1970).

22 Nieuwkoop, P. D. & Suminski, E. H. Does the so-called "germinal plasm" play an important role in the development of the primordial germ cells. *Arch. Anat. Microsc. Morphol. Exp.* **48**, 189-198 (1959).

23 Ikenishi, K., Kotani, M. & Tanabe, K. Ultrastructural changes associated with UV irradiation in the "germinal plasm" of *Xenopus laevis*. *Dev. Biol.* **36**, 155-168 (1974).

24 Tanabe, K. & Kotani, M. Relationship between the amount of the "germinal plasm" and the number of primordial germ cells in *Xenopus laevis*. *J. Embryol. Exp. Morphol.* **31**, 89-98 (1974).

25 Züst, B. & Dixon, K. E. The effect of U.V. irradiation of the vegetal pole of *Xenopus laevis* eggs on the presumptive primordial germ cells. *J. Embryol. Exp. Morphol.* **34**, 209-220 (1975).

26 Ikenishi, K., Nakazato, S. & Okuda, T. Direct Evidence for the Presence of Germ Cell Determinant in Vegetal Pole Cytoplasm of *Xenopus laevis* and in a Subcellular Fraction of It. *Development, Growth and Differentiation* **28**, 563-568 (1986).

27 Falin, L. I. The development of genital glands and the origin of germ cells in human embryogenesis. *Acta Anat (Basel)* **72**, 195-232 (1969).

28 Simkins, C. S. Origin of the germ cells in Man. *American Journal of Anatomy* **41**, 249-293 (1928).

29 Witschi, E. Migration of the germ cells of human embryos from the yolk sac to the primitive gonadal folds. *Contributions to Embryology* **209**, 67-80 (1948).

30 Christophers. (Cambridge University Press, 1960).

31 Vangestel, S., Houthoofd, W., Bert, W. & Borgonie, G. The early embryonic development of the satellite organism *Pristionchus pacificus*: differences and similarities with *Caenorhabditis elegans*. *Nematology* **10**, 301-312 (2008).

32 Dolinski, C., Baldwin, J. G. & Thomas, W. K. Comparative survey of early embryogenesis of Secernentea (Nematoda), with phylogenetic implications. *Can. J. Zool.* **79**, 82-94 (2001).

33 Toshiki, T. *et al.* Germline transformation of the silkworm *Bombyx mori* L. using a piggyBac transposon-derived vector. *Nature Biotech.*, 81-84 (2000).

34 Miya, K. Studies on the embryonic development of the gonad in the silkworm, *Bombyx mori* L. Part I. Differentiation of germ cells. *Journal of the Faculty of Agriculture of Iwate University* **3**, 436-467 (1958).

35 Miya, K. Ultrastructural changes of embryonic cells during organogenesis in the silkworm, *Bombyx mori*. I. The Gonad. *Journal of the Faculty of Agriculture of Iwate University* **12**, 329-338 (1975).

36 Miya, K. The presumptive genital region at the blastoderm stage of the silkworm egg. *Journal of the Faculty of Agriculture of Iwate University*, 223-227 (1953).

37 Tomaya, K. On the embryology of the silkworm. *Bull. Coll. Agriculture, Tokyo* **5**, 73-111 (1902).

38 Nakao, H. Isolation and characterization of a *Bombyx vasa*-like gene. *Dev. Genes Evol.* **209**, 312-316 (1999).

39 Nakao, H., Hatakeyama, M., Lee, J. M., Shimoda, M. & Kanda, T. Expression pattern of *Bombyx* vasa-like (BmVLG) protein and its implications in germ cell development. *Dev. Genes Evol.* **216**, 94-99 (2006).

40 Hird, S. N., Paulsen, J. E. & Strome, S. Segregation of germ granules in living *Caenorhabditis elegans* embryos: cell-type-specific mechanisms for cytoplasmic localisation. *Development* **122**, 1303-1312 (1996).

41 Deppe, U. *et al.* Cell lineages of the embryo of the nematode *Caenorhabditis elegans*. *Proc. Natl. Acad. Sci. USA* **75**, 376-380 (1978).

42 Strome, S. & Wood, W. B. Immunofluorescence visualization of germ-line-specific cytoplasmic granules in embryos, larvae, and adults of *Caenorhabditis elegans*. *Proc. Natl. Acad. Sci. USA* **79**, 1558-1562 (1982).

43 Meyer, N. P., Boyle, M. J., Martindale, M. Q. & Seaver, E. C. A comprehensive fate map by intracellular injection of identified blastomeres in the marine polychaete Capitella teleta. *EvoDevo* **1**, 8, doi:10.1186/2041-9139-1-8 (2010).

44 Dill, K. K. & Seaver, E. C. *vasa* and *nanos* are coexpressed in somatic and germ line tissue from early embryonic cleavage stages through adulthood in the polychaete *Capitella sp. I*. *Dev. Genes Evol.* **218**, 453-463 (2008).

45 Giani, V. C., Emi, Y., Michael, B. J. & Seaver, E. C. Somatic and germline expression of *piwi* during development and regeneration in the marine polychaete annelid *Capitella teleta*. *EvoDevo* **2**, 10, doi:10.1186/2041-9139-2-10 (2011).

46 Oelhafen, F. Zur embryogenese von *Culex pipiens*: Markierungen und exstirpationen mit UV-strahlenstich. *Roux' Archiv für Entwicklungsmechanik* **153**, 120-157 (1961).

47 Sagawa, K., Yamagata, H. & Shiga, Y. Exploring embryonic germ line development in the water flea, *Daphnia magna*, by zinc-finger-containing VASA as a marker. *Gene Expression Patterns* **5**, 669-678 (2005).

48 Kang, D., Pilon, M. & Weisblat, D. A. Maternal and zygotic expression of a *nanos*-class gene in the leech *Helobdella robusta*: primordial germ cells arise from segmental mesoderm. *Dev. Biol.* **245**, 28-41 (2002).

49 Cho, S. J., Vallès, Y. & Weisblat, D. A. Differential expression of conserved germ line markers and delayed segregation of male and female primordial germ cells in a hermaphrodite, the leech helobdella. *Mol. Biol. Evol.* **31**, 341-354, doi:10.1093/molbev/mst201 (2014).

50 Landmann, F. *et al.* Both asymmetric mitotic segregation and cell-to-cell invasion are required for stable germline transmisison of Wilbachia in filarial nematodes. *Biol Open* **1**, 536-547 (2012).

51 Marinotti, O. *et al.* The genome of Anopheles darlingi, the main neotropical malaria vector. *Nucleic Acids Res.* **41**, 7387-7400, doi:10.1093/nar/gkt484 (2013).

52 Neafsey, D. E. *et al.* Mosquito genomics. Highly evolvable malaria vectors: the genomes of 16 Anopheles mosquitoes. *Science* **347**, 1258522, doi:10.1126/science.1258522 (2015).

53 Zhan, X. *et al.* Peregrine and saker falcon genome sequences provide insights into evolution of a predatory lifestyle. *Nat. Genet.* **45**, 563-566, doi:10.1038/ng.2588 (2013).

54 Ellegren, H. The Evolutionary Genomics of Birds. *Annual Review Of Ecology And Systematics* **44**, 239-259 (2013).

55 Green, R. E. *et al.* Three crocodilian genomes reveal ancestral patterns of evolution among archosaurs. *Science* **346**, 1254449, doi:10.1126/science.1254449 (2014).

56 Eberle, J. J., Gottfried, M. D., Hutchison, J. H. & Brochu, C. A. First record of eocene bony fishes and crocodyliforms from Canada's Western Arctic. *PLoS ONE* **9**, e96079, doi:10.1371/journal.pone.0096079 (2014).

57 Zhang, G., Li, C., Li, A. & Li, B. Comparative genomics reveals insights into avian genome evolution and adaptation. *Science* **346**, 1311-1320 (2014).

58 Sharma, A. & Federico, G. in *Mid-South Computational Biology and Bioinformatics Society (MCBIOS) Conference: Making Sense of the Omics Data Deluge* (2012).

59 Rocha, E. P. *et al.* Comparisons of dN/dS are time dependent for closely related bacterial genomes. *J. Theor. Biol.* **239**, 226-235, doi:10.1016/j.jtbi.2005.08.037 (2006).

60 Cutter, A. D. Divergence times in Caenorhabditis and Drosophila inferred from direct estimates of the neutral mutation rate. *Mol. Biol. Evol.* **25**, 778-786, doi:10.1093/molbev/msn024 (2008).

61 Angelini, D. R. & Jockusch, E. L. Relationships among pest flour beetles of the genus Tribolium (Tenebrionidae) inferred from multiple molecular markers. *Mol. Phylogenet. Evol.* **46**, 127-141, doi:10.1016/j.ympev.2007.08.017 (2008).

62 Campbell, B. C., Steffen-Campbell, J. D. & Werren, J. H. Phylogeny of the Nasonia species complex (Hymenoptera: Pteromalidae) inferred from an internal transcribed spacer (ITS2) and 28S rDNA sequences. *Insect Mol. Biol.* **2**, 225-237 (1993).

63 Oliveira, D. C., Raychoudhury, R., Lavrov, D. V. & Werren, J. H. Rapidly evolving mitochondrial genome and directional selection in mitochondrial genes in the parasitic wasp nasonia (hymenoptera: pteromalidae). *Mol. Biol. Evol.* **25**, 2167-2180, doi:10.1093/molbev/msn159 (2008).

64 Engel, M. S. A giant honey bee from the Middle Miocene of Japan (Hymenoptera : Apidae). *American Museum Novitates* **3504**, 1-12 (2006).

65 Stone, A. C. *et al.* More reliable estimates of divergence times in Pan using complete mtDNA sequences and accounting for population structure. *Philos Trans R Soc Lond, B, Biol Sci* **365**, 3277-3288 (2010).

66 Hellsten, U. *et al.* Accelerated gene evolution and subfunctionalization in the pseudotetraploid frog Xenopus laevis. *BMC biology* **5**, 31, doi:10.1186/1741-7007-5-31 (2007).

67 Subramanian, S. Significance of population size on the fixation of nonsynonymous mutations in genes under varying levels of selection pressure. *Genetics* **193**, 995-1000 (2013).

68 Wan, Q. H. *et al.* Genome analysis and signature discovery for diving and sensory properties of the endangered Chinese alligator. *Cell Research* **23**, 1091-1105 (2013).

69 Ohta, T. Evolutionary rate of cistrons and DNA divergence. *J. Mol. Evol.* **1**, 150-157 (1972).

70 Evans, T., Wade, C. M., Chapman, F. A., Johnson, A. D. & Loose, M. Acquisition of germ plasm accelerates vertebrate evolution. *Science* **344**, 200-203, doi:10.1126/science.1249325 (2014).

71 Duret, L. & Mouchiroud, D. Expression pattern and, surprisingly, gene length shape codon usage in Caenorhabditis, Drosophila, and Arabidopsis. *Proc. Natl. Acad. Sci. USA* **96**, 4482-4487 (1999).

72 Vicario, S., Moriyama, E. N. & Powell, J. R. Codon usage in twelve species of Drosophila. *BMC evolutionary biology* **7**, 226, doi:10.1186/1471-2148-7-226 (2007).

73 Williford, A. & Demuth, J. P. Gene expression levels are correlated with synonymous codon usage, amino acid composition, and gene architecture in the red flour beetle, *Tribolium castaneum*. *Mol. Biol. Evol.* **29**, 3755-3766, doi:10.1093/molbev/mss184 (2012).

74 Mueller, J. L. *et al.* Cross-species comparison of Drosophila male accessory gland protein genes. *Genetics* **171** (2005).

75 Plotkin, J. B., Dushoff, J., Desai, M. M. & Fraser, H. B. Estimating selection pressures from limited comparative data. *Mol. Biol. Evol.* **23**, 1457-1459 (2006).

76 Schmid, K. J. & Aquadro, C. F. The evolutionary analysis of "orphans" from the Drosophila genome identifies rapidly diverging and incorrectly annotated genes. *Genetics* **159**, 589-598 (2001).

77 Haddrill, P. R., Zeng, K. & Charlesworth, B. Determinants of synonymous and nonsynonymous variability in three species of Drosophila. *Mol. Biol. Evol.* **28**, 1731-1743, doi:10.1093/molbev/msq354 (2011).

78 Ran, W., Kristensen, D. M. & Koonin, E. V. Coupling Between Protein Level Selection and Codon Usage Optimization in the Evolution of Bacteria and Archaea. *mBio* **5**, e00956-00914, doi:10.1128/mBio.00956-14 (2014).

79 Comeron, J. M. & Kreitman, M. The Correlation Between Synonymous and Nonsynonymous Substitutions in *Drosophila*: Mutation, Selection or Relaxed Constraints? *Genetics*, 767-775 (1998).

80 Drummond, D. A. & Wilke, C. O. Mistranslation-induced protein misfolding as a dominant constraint on coding-sequence evolution. *Cell* **134**, 341-352 (2008).

81 Sella, G., Petrov, D. A., Przeworski, M. & Andolfatto, P. Pervasive natural selection in the Drosophila genome? *PLoS genetics* **5**, e1000495, doi:10.1371/journal.pgen.1000495 (2009).

82 Betancourt, A. J. & Presgraves, D. C. Linkage limits the power of natural selection in Drosophila. *Proc. Natl. Acad. Sci. USA* **99**, 13616-13620, doi:10.1073/pnas.212277199 (2002).

83 Kim, Y. Effect of strong directional selection on weakly selected mutations at linked sites: implication for synonymous codon usage. *Mol. Biol. Evol.* **21**, 286-294, doi:10.1093/molbev/msh020 (2004).

84 Pollard, D. A., Iyer, V. N., Moses, A. M. & Eisen, M. B. Widespread discordance of gene trees with species tree in Drosophila: evidence for incomplete lineage sorting. *PLoS genetics* **2**, e173, doi:10.1371/journal.pgen.0020173 (2006).

85 Kline, R. B. *Principles and Practice of Structural Equation Modeling*. 2nd edn, 366 (Guilford Press, 2004).

86 Wang, B. *et al.* Optimal codon identities in bacteria: implications from the conflicting results of two different methods. *PLoS ONE* **6**, e22714 (2011).

87 Wright, F. The "effective number of codons" used in a gene. *Gene* **87**, 23-29 (1990).

88 Sharp, P. M. & Li, W.-H. An evolutionary perspective on synonymous codon usage in unicellular organisms. *J. Mol. Evol.* **24**, 28-38 (1986).

89 Whittle, C. A., Sun, Y. & Johannesson, H. Evolution of synonymous codon usage in *Neurospora tetrasperma* and *Neurospora discreta*. *Genome Biol Evol* **3**, 332-343 (2011).

90 Supek, F., Skunca, N., Repar, J., Vlahovicek, K. & Smuc, T. Translational selection is ubiquitous in prokaryotes. *PLoS Genetics* **6**, e1001004 (2010).

91 Carlini, D. B. & Makowski, M. Codon bias and gene ontology in holometabolous and hemimetabolous insects. *Journal of Experimental Zoology Part B: Molecular and Developmental Evolution*, doi:doi: 10.1002/jez.b.22647 (2015).

92 Stein, L. D. *et al.* The genome sequence of Caenorhabditis briggsae: A platform for comparative genomics. *PLoS Biol.* **1**, 166-192 (2003).

93 Wei, L. *et al.* Analysis of codon usage bias of mitochondrial genome in Bombyx mori and its relation to evolution. *BMC evolutionary biology* **14**, 262 (2014).

94 Jia, X. *et al.* Non-uniqueness of factors constraint on the codon usage in *Bombyx mori*. *BMC Genomics* **16**, 356, doi:10.1186/s12864-015-1596-z (2015).

95 Webster, B. L., Southgate, V. R. & Littlewood, D. T. A revision of the interrelationships of Schistosoma including the recently described Schistosoma guineensis. *Int. J. Parasitol.* **36**, 947-955, doi:10.1016/j.ijpara.2006.03.005 (2006).

96 Nakao, M., Lavikainen, A., Yanagida, T. & Ito, A. Phylogenetic systematics of the genus Echinococcus (Cestoda: Taeniidae). *Int. J. Parasitol.* **43**, 1017-1029, doi:10.1016/j.ijpara.2013.06.002 (2013).

97 *Encyclopedia of Life*, <http://www.eol.org> (2014).

98 Lebedinski, J. Die Entwicklung der Daphnia aus dem Sommerei. *Zool. Anz.* **14** (1891).

99 Kaudewitz, F. Zur Entwicklungsphysiologie von *Daphnia pulex*. *Roux' Archiv für Entwicklungsmechanik* **144**, 410-447 (1950).

100 Forró, L., Korovchinsky, N. M., Kotov, A. A. & Petrusek, A. Global diversity of cladocerans (Cladocera; Crustacea) in freshwater. *Hydrobiol.* **595**, 177-184 (2008).

101 Miura, T. *et al.* A comparison of parthenogenetic and sexual embryogenesis of the pea aphid *Acyrthosiphon pisum* (Hemiptera: Aphidoidea). *J Exp Zool* **295B**, 59-81 (2003).

102 Metschnikoff, E. Embryologische Studien an Insekten. *Zeit. f. wiss Zool.* **16**, 389-500 (1866).

103 Witlaczil, E. Entwicklungsgeschichte der Aphiden. *Zeit. f. wiss Zool.* **40**, 559-690 (1884).

104 Will, L. Entwicklungsgescshichte der viviparen Aphiden. *Zool. Jarh.* **3**, 201-280 (1888).

105 Capinera, J. L. 4346 (Springer, Germany, 2008).
